# Understanding divergent domestication traits from the whole-genome sequencing of swamp- and river-buffalo populations

**DOI:** 10.1093/nsr/nwaa024

**Published:** 2020-02-17

**Authors:** Xier Luo, Yu Zhou, Bing Zhang, Yi Zhang, Xiaobo Wang, Tong Feng, Zhipeng Li, Kuiqing Cui, Zhiqiang Wang, Chan Luo, Hui Li, Yanfei Deng, Fenghua Lu, Jianlin Han, Yongwang Miao, Huaming Mao, Xiaoyan Yi, Cheng Ai, Shigang Wu, Alun Li, Zhichao Wu, Zijun Zhuo, Do Da Giang, Bikash Mitra, Mohammad Farhad Vahidi, Shahid Mansoor, Sahar Ahmed Al-Bayatti, Eka Meutia Sari, Neena Amatya Gorkhali, Sigit Prastowo, Laiba Shafique, Guoyou Ye, Qian Qian, Baoshan Chen, Deshun Shi, Jue Ruan, Qingyou Liu

**Affiliations:** 1 State Key Laboratory for Conservation and Utilization of Subtropical Agro-Bioresources, Guangxi University, Nanning 530005, China; 2 Guangdong Laboratory for Lingnan Modern Agriculture, Genome Analysis Laboratory of the Ministry of Agriculture, Agricultural Genomics Institute at Shenzhen, Chinese Academy of Agricultural Sciences, Shenzhen 518120, China; 3 CAS Key Laboratory of Genome Sciences and Information, Beijing Institute of Genomics, Chinese Academy of Sciences, Beijing 100101, China; 4 National Engineering Laboratory for Animal Breeding, Key Laboratory of Animal Genetics, Breeding and Reproduction of Ministry of Agriculture and Rural Affairs, College of Animal Science and Technology, China Agricultural University, Beijing 100083, China; 5 CAAS-ILRI Joint Laboratory on Livestock and Forage Genetic Resources, Institute of Animal Science, Chinese Academy of Agricultural Sciences, Beijing 100193, China; 6 Faculty of Animal Science and Technology, Yunnan Agricultural University, Kunming 650201, China; 7 Bacgiang Agriculture and Forestry University, Bacgiang 230000, Vietnam; 8 Cellular Immunology Lab, Department of Zoology, University of North Bengal, Siligun 734013, India; 9 Animal Biotechnology Department, Agricultural Biotechnology Research Institute of Iran-North Region, Agricultural Research, Education and Extension Organization, Rasht 999067, Iran; 10 National Institute for Biotechnology and Genetic Engineering, Faisalabad 999010, Pakistan; 11 Animal Genetic Sources Department, Directorate of Animal Resources, Ministry of Agriculture, Baghdad 19207, Iraq; 12 Department of Animal Science, Faculty of Agriculture, Syiah Kuala University, Darussalam-Banda Aceh 23111, Indonesia; 13 Animal Breeding Division, National Animal Science Research Institute, Nepal Agriculture Research Council, Khumaltar 999098, Nepal; 14 Animal Science Department Universitas Sebelas Maret, Surakarta 999006, Indonesia; 15 International Rice Research Institute, Manila 999005, Philippines; 16 International Livestock Research Institute, Nairobi 00100, Kenya

**Keywords:** *Bubalus bubalis* genome, buffalo, domestication, re-sequencing, traits

## Abstract

Domesticated buffaloes have been integral to rice-paddy agro-ecosystems for millennia, yet relatively little is known about the buffalo genomics. Here, we sequenced and assembled reference genomes for both swamp and river buffaloes and we re-sequenced 230 individuals (132 swamp buffaloes and 98 river buffaloes) sampled from across Asia and Europe. Beyond the many actionable insights that our study revealed about the domestication, basic physiology and breeding of buffalo, we made the striking discovery that the divergent domestication traits between swamp and river buffaloes can be explained with recent selections of genes on social behavior, digestion metabolism, strengths and milk production.

## INTRODUCTION

The water buffalo is a globally important domestic animal of immense worth to humans, providing economic value from milk, meat, leather and especially draft power [1]. Worldwide population estimates of more than 200 million head are relied on by more than 2 billion people—more than any other domesticated animal [2]. Most buffaloes work on small farms in close association with humans, for whom these animals are often their greatest capital asset. Buffaloes are even-toed, hoofed mammals of the Bovidae family, Genus Bubalus and tribe Bovini. There are two sub-species of the domesticated water buffalo that are thought to have undergone independent domestication processes [3,4]: swamp buffalo (*Bubalus bubalis carabanesis*, 2 N = 48) and river buffalo (*Bubalus bubalis bubalis*, 2 N = 50).

The distribution of swamp buffaloes overlaps closely with rice agriculture in East and Southeast Asian countries (e.g. China, Vietnam, Thailand, etc.), where they have served as the primary draft animals for rice cultivation over thousands of years. Archaeological excavation of the Hemudu Cultural Site in present-day Zhejiang Province, China found 150 tons of rice and 170 pieces of agricultural equipment made of buffalo scapula from 7000 years ago, indicating the presence of a highly developed agrarian interdependence among humans, buffaloes and rice by this time in history. Domesticated swamp buffaloes are extraordinarily docile and obedient; even young children can rein them easily, with exceedingly few reports of attacks on humans by swamp buffaloes [5].

River buffaloes are mainly distributed in India, Pakistan, the Middle East and Italy, and are prized for their distinctive, high-quality milk typified by high-fat and dry-matter content. Buffalo milk has lower cholesterol but more calories and fat than cow’s milk, and produces high-value, thick and creamy cheeses, yoghurt, khoa and ghee [6]. In contrast to swamp buffaloes, river buffaloes exhibit increased aggression and temperamental instability [7]. Modern mechanization has lessened the need for buffalo-based tillage in all but the steepest paddy terraces, resulting in attempts to introduce milk-producing river buffaloes where swamp buffaloes were traditionally favored. However, the more aggressive behavior of river buffaloes and swamp/river hybrids has limited the success of such efforts.

Extensive research into the breeding and development of hybrid buffaloes has been aided by advances in applied and molecular genetics. However, even with the sequencing of the river buffalo [8–10] and the wild African water buffalo [11] (*Syncerus caffer*) genomes, buffalo-genome resources are still insufficient to enable researchers and breeders to leverage the modern tools and approaches that could facilitate major basic and applied advances (Supplementary Table 1). Here, we generated two high-quality reference genomes for swamp and river buffaloes based on multiple sequencing and assembly efforts. In addition to examining chromosomal fusions and other structural variations in the genomes of these buffalo sub-species, we conducted a large-scale re-sequencing analysis of 230 individuals (132 swamp buffaloes and 98 river buffaloes) from across Asia and Europe. We then performed a variety of demography, population-structure and selective-sweep analyses to substantially extend our understanding of the genetic and physiological diversity and evolutionary history of these hugely important animals. Most strikingly, we detected swamp-buffalo-specific selection for genes related to brain development and cognition, potentially helping to explain the genetic basis of the docility that enables these animals to act as long-term collaborators in the development of rice-paddy agro-ecosystems. In addition, a variety of river-buffalo-specific selected sweep genes related to fecundity, milk production and body size were identified, which was beneficial to the comprehensive practice in selection and breeding combined with genome-wide association studies (GWAS).

## RESULTS

### Assembly and annotation of reference genomes for swamp buffaloes and river buffaloes

We combined multiple approaches to sequence and assemble the swamp-buffalo and river-buffalo reference genomes. DNA was sequenced from one female Fuzhong swamp buffalo and one female Murrah river buffalo (Supplementary Figs 1–3) and resulting PacBio assemblies were built into contig N50 sizes of 8.8 and 3.1 Mb for swamp and river buffaloes, respectively. These contigs were first scaffolded, then clustered using Illumina High-throughput/resolution chromosome conformation capture sequencing (Hi-C) into chromosome-scale scaffolds with N50 sizes of 117.3/116.1 Mb, the longest being 269.09/198.8 Mb for swamp/river buffaloes (Table 1, Supplementary Tables 2–6 and Supplementary Figs 4–11). The final assemblies comprised 24 chromosomes covering 97.5% of the swamp-buffalo genome and 25 chromosomes covering 96.5% of the river-buffalo genome, and assessments of nucleotide accuracy showed that the error rate was 9.22 × 10^−6^ in swamp buffaloes and 2.13 × 10^−5^ in river buffaloes (Supplementary Table 7).

**Table 1.** Summary statistics for the genome sequences, annotation and population parameters.

		Swamp buffalo	River buffalo
Genome	Total genome size (Mb)	2631	2646
	Chromosomes number	24	25
	Scaffolds number^a^	24 + 1510	25 + 2279
	Scaffolds N50 (Mb)	117.3	116.1
	Scaffolds L50	8	9
	Total contigs size (Mb)	2609	2626
	Contigs N50 (Mb)	8.8	3.1
Annotation	Total gene number	19 279	20 202
	Average CDS length (bp)	1764.5	1662.2
	BUSCO assessment	96.80%	96.00%
	Repeat content	47.26%	47.31%
Population	Sample number	132	98
	SNP number^b^	18 737 564	23 722 820
	Genetic diversity *θ* (4Nμ)	0.001805	0.002743
	Population differentiation (*F*_st_)	0.27	

^a^Number of chromosome-level scaffolds and unplaced scaffolds.

^b^SNPs called among samples without potential gene flow.

**Figure 1.**
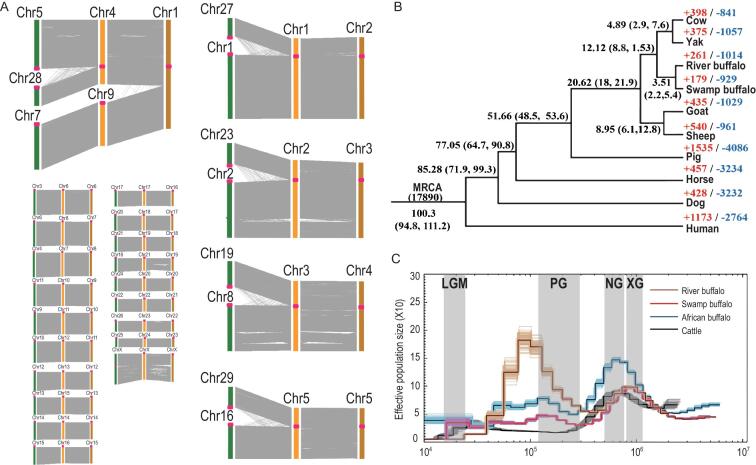
(A) Synteny analysis among cattle, swamp buffaloes and river buffaloes. The colored bars represent the chromosome of cattle (green), river buffaloes (orange) and swamp buffaloes (brown). The red dots represent the position of the centromere in the chromosome. (B) A phylogenetic tree generated using single-copy orthologous genes. Numbers on the nodes are the divergence time from the present (Mya) and its confidence interval. The number of expanded (red) and contracted (blue) gene families are shown on the species name. MRCA, most recent common ancestor. (C) Demographic history inferred by PSMC. We used a generation time of 6 years for buffaloes and 5 years for cattle. The mutation rate was 2.2 × 10^–9^ mutations per nucleotide per year for all samples. The period of the Xixiabangma Glaciation (XG, 1.1–0.8 Ma), the Naynayxungla Glaciation (NG, 0.78–0.5 Ma) and the Penultimate Glaciation (PG, 0.30–0.13 Ma) and the last glacial maximum (LGM, ∼20 kya) are shaded in gray.

The mapping rate and coverage from Illumina reads reached >99% in both buffaloes (Supplementary Table 8). Repetitive sequences comprise 46.62%/46.37% of the swamp/river-buffalo genomes (Table 1), with a similar proportion of transposable element classes and ruminant-specific repeats as reported for cattle (*Bos taurus*). Previous studies suggest that chromosomal fusions have potentially contributed to the speciation of buffaloes [12] and it is well established that hybrids (2 N = 49) resulting from crosses between swamp buffaloes (2 N = 48) and river buffaloes (2 N = 50) can be fertile but exhibit reduced fecundity. We therefore used our reference genomes to investigate chromosomal rearrangements in cattle (2 N = 60), swamp buffaloes and river buffaloes; this comparison of sequence synteny identified five chromosome-fusion events between cattle and river buffaloes (yielding five bi-armed autosome pairs). Specifically, chromosomes 4 and 9 of river buffaloes are linked, representing the largest chromosome in swamp buffaloes (Fig. 1A). Our analysis also confirmed previous findings from karyotype studies about the orientation and order of the sequences within fusion chromosomes [13]. Also note that we detected no obvious gene-fusion events at chromosome-fusion breakpoints.

Using repeat-masked genomes, we identified 19 279/20 202 gene models in the swamp/river-buffalo genomes. BUSCO assessments indicated that the genomes were 96.8%/96.0% complete, underscoring the high quality of the genomes and the gene-structure predictions (Table 1, Supplementary Tables 9–12 and Supplementary Fig. 12). An OrthoMCL analysis of the 10 mammal taxa (swamp buffalo, river buffalo, human, cattle, yak, mouse, dog, horse, sheep and goat) identified 17 890 gene families. To estimate the divergent time between swamp buffaloes and river buffaloes, we performed Ks-based age-distribution and Bayesian estimation under a molecular-clock model using 6429 single-copy orthologs and the analysis showed that swamp buffaloes and river buffaloes shared a common ancestor 1.1–3.5 million years ago (Fig. 1B and Supplementary Fig. 13). By applying a branch-site model and likelihood-ratio tests, we detected 99 and 78 positive selection genes for swamp and river buffaloes (Supplementary Table 13).

Compared to the *Bos genus*, *Bubalus* showed greater events of gene-family contraction (2117 compared to 1022) and fewer events of gene-family expansion (112 versus 148). Moving from gene families to the gene level, we also identified 179 and 261 of swamp- and river-specific expanded genes (Supplementary Tables 14–16 and Supplementary Figs 14–18), which may relate to the different phenotypes of the two buffalo sub-species. For instance, the *AMD1* gene of polyamine biosynthesis was significantly expanded in swamp buffaloes: there are three *AMD1* paralogues in swamp buffaloes but only one both in river buffaloes and cattle (Supplementary Figs 19 and 20). *AMD1* encodes for the S-adenosylmethionine decarboxylase that is essential in the biosynthesis of polyamines (spermidine and spermine) and the upregulation of *AMD1* caused polyamine accumulation in muscle tissues, thereby promoting muscle growth, suggesting that a higher *AMD1* copy number may help to explain the muscular-physique characteristic of swamp buffaloes [14]. The heat-shock protein *Hsp90A* subfamily expanded in both buffalo sub-species. It encodes heat-shock protein 90α (*Hsp90α*), a stress-inducible molecular chaperone, which is related to adapting to environmental stress (Supplementary Fig. 21).

### Demography analysis of buffaloes and other bovines

We used a pairwise sequentially Markovian coalescent (PSMC) analysis to examine changes in the effective population size (*N*_e_) of the ancestral populations of swamp/river buffaloes, cattle and the wild African buffalo (*Syncerus caffer*) (Fig. 1C). Each of the four populations experienced two expansions and two contractions in their *N*_e_. Swamp buffaloes and river buffaloes declined at ∼0.8 Ma; however, the ancestor of river buffaloes experienced a relatively shorter bottleneck with a rapid ascent of *N*_e_ at 0.3 Ma with a peak at 0.1 Ma, whereas the ancestor of swamp buffaloes only ascended slightly at 0.3 Ma.

It is worth mentioning the striking peak of effective population size at 0.1 Ma in river buffaloes. The fluctuations of *N*_e_ in the two buffalo sub-species coincided with previously reported fluctuations of *N*_e_ in the taurine and indicine sub-species of cattle during the largest Pleistocene glaciations [15]. River buffaloes and indicine cattle had the same geographical distribution in South Asia. The striking peak of *N*_e_ about Bovinae was most likely caused by habitat expansion or migration hybridization, when the sea level depressed in the Penultimate Glacial (0.13 ∼ 0.30 Ma) [16–18].

### Re-sequencing of geographically diverse swamp- and river-buffalo populations

**Figure 2.**
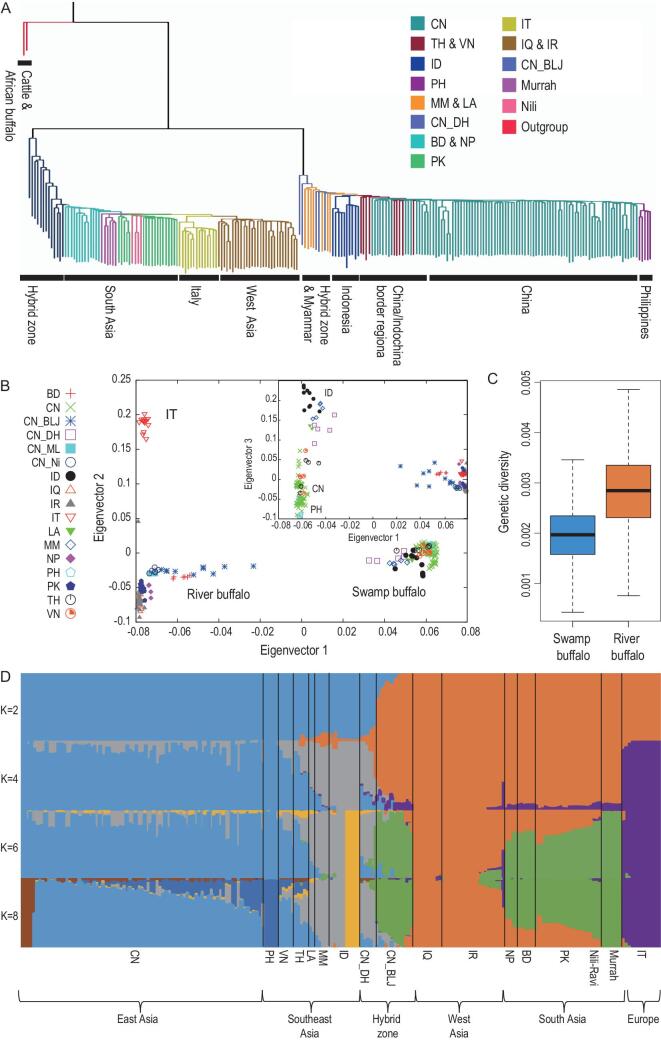


To investigate the population features amongst domesticated buffalo populations, we re-sequenced 132 swamp-buffalo and 98 river-buffalo individuals from 15 countries—China, Vietnam, Thailand, Laos, Myanmar, Indonesia, India, the Philippines, Pakistan, Nepal, Bengal, Iran, Iraq, Bengal and Italy—with 5–10× coverage depth (based on WGS short-read data) (Fig. 2A). This population-diversity panel covered individuals spanning most countries with domesticated buffaloes (Supplementary Table 17). All sequencing data were aligned against both genomes to facilitate the identification of small genomic variants and, after applying a minor allele-frequency method (MAF >2.5%), we identified 18 732 366 SNPs for the swamp-buffalo population, 23 722 820 SNPs for the river-buffalo population and 33 516 506 SNPs for both sub-species of buffaloes. Most individuals had >98% genome coverage and >98% mapping rates for both the swamp-buffalo and river-buffalo reference genomes, thereby allowing high-confidence variant calling (Supplementary Tables 18 and 19).

**Figure 2.** Population structure and genetic diversity of buffaloes. (A) A neighbor-joining phylogenetic tree constructed using whole-genome SNPs data. Out-groups: cattle and African buffaloes. (B) Principal component analysis (PCA) plots of the first three components. Outset was between PC1 and PC2. Inset was between PC1 and PC3. CN, China; TH, Thailand; VN, Vietnam; ID, Indonesia; PH, Philippines; MM, Myanmar; LA, Laos; CN_DH, CN_BLJ, buffaloes from hybrid area of China; BD, Bengal; NP, Nepal; PK, Pakistan; IT, Italy; IQ, Iraq; IR, Iran. (C) Genetic diversity (θ, 4Nμ) for buffalo populations. (D) Population-structure plots with K = 2 ∼ 8. The *y*-axis quantifies the proportion of the individual's genome from inferred ancestral populations and the *x*-axis shows the different populations clustered by countries or districts.

### Population structure and genetic diversity

In subsequent analyses of re-sequencing data, SNPs present on X chromosomes were excluded to avoid potential bias by sex. A principal component analysis (PCA) revealed that the swamp-buffalo and river-buffalo individuals are clearly separated into distinct clusters. A phylogenetic tree reconstructed using a neighbor-joining (NJ) approach also supports this clear separation and individuals sampled from the hybrid zone were positioned close to the ancestral nodes for both sub-species of buffalo [19,20] (Fig. 2A and B and Supplementary Figs 22–24).

Furthermore, we conducted an analysis of population structures using an expectation-maximization (EM) algorithm that clusters individuals from specified countries (or districts) into K number of ancestral populations. When K = 2, the majority of the swamp and river buffaloes were clearly split into two groups. However, even at this low K number, it was clear that swamp buffaloes in Laos (LA), Myanmar (MM) and Yunnan Province of China (CN_DH) possessed genetic components from river buffaloes and that river buffaloes in Yunnan Province of China (CN_BLJ) had genetic components from swamp buffaloes. When K = 4, there were two obvious groups of swamp buffaloes: one from the Philippines and China, and the other from Indonesia. There were also two obvious groups of river buffaloes: one for the Italian individuals and one containing the remaining river buffaloes (including those from South Asia and the Middle East). When K = 6, the Indonesian swamp buffaloes from two different islands were split into two groups and river buffaloes were divided between South Asia and the Middle East. When K = 8, additional groups were apparent amongst the Philippine and Chinese as well as the inner structure of Chinese swamp buffaloes (Fig. 2D and Supplementary Fig. 25).

Seeking to infer the history of divergence and admixture among populations, we used Treemix [21] to assess the joint allele frequencies for both buffalo sub-species from each country (or district). Two buffalo sub-species were mutually selected as the out-group. When this analysis was conducted without allowing migration tracks, we obtained the same topological structure as in the aforementioned phylogenetic analysis. If migration tracks were allowed, there was strong support for migration events in both the swamp buffaloes (MM and CN_DH) and river buffaloes (CN_BLJ, NP and BD) (Supplementary Fig. 26). Patterns of these migrations may have shaped the apparent hybrid zone from which we sampled individuals harboring a genomic admixture of swamp and river buffaloes. This hypothetical scenario corresponds to our phylogeny, indicating high gene flow amongst buffaloes in certain Southeast Asian regions. Targeted sampling organized around local topographical features could test the hypothesis about the role of migration patterns in shaping the population genetics of buffaloes. We also analysed genetic diversity *θ*π (4Nμ): after removing individuals with >5% admixture, river buffaloes showed overall higher genetic diversity than swamp buffaloes (Fig. 2C and Supplementary Fig. 27). We noted that, on a country-by-country basis, Italian buffaloes had a particularly low *θ*π value. Furthermore, the *θ*π values for the Murrah- and Nili-Ravi-buffalo individuals from China were much lower than those from Pakistan, which can likely be explained by the official introduction of Chinese river-buffalo progenitors from India and Pakistan to the Chinese government in 1957 and 1974. That is, this reduced genetic diversity for Chinese river buffaloes is entirely consistent with the known severe genetic bottleneck that they underwent in the mid-twentieth century [6].

### Signals of recent selective sweep in both buffalo sub-species

**Figure 3.**
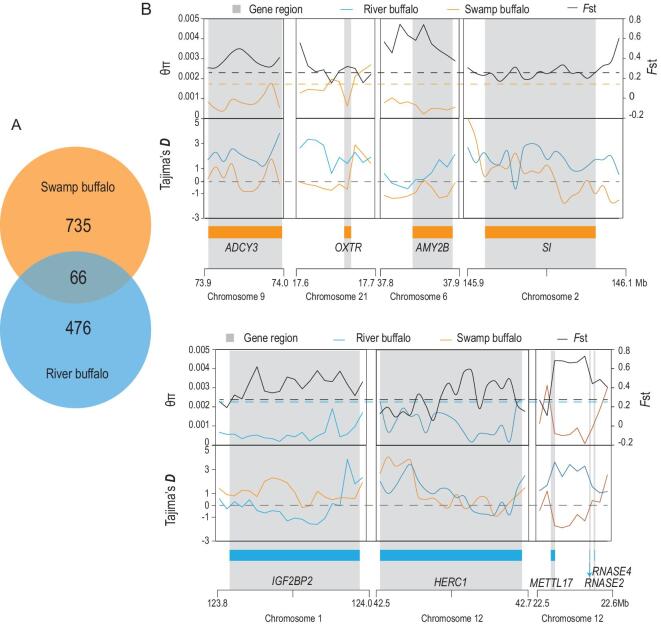
Sweep-gene sets between swamp buffaloes and river buffaloes show the divergent domestication direction respectively. (A) Venn-like diagram presenting the selective-sweep genes between swamp buffaloes and river buffaloes. (B) Example of genes with strong selective-sweep signals in swamp buffaloes. (C) Example of genes with strong selective-sweep signals in river buffaloes. The *θ*π, *F*st and Tajima's *D* values were plotted with a 10-kb sliding window. The dark horizontal dotted lines represent the mean value of *F*st; the orange horizontal dotted lines represent the mean value of *θ*π; the gray at the range horizontal dotted lines represent the value of zero.

To identify genomic regions harboring artificial selection signatures in the swamp and river buffaloes, we used Sweepfinder2 to scan for selective sweeps using the spatial distribution of allele frequencies, the integrated haplotype score method (iHS) to measure extended haplotype homozygosity (EHH) at a given SNP along the ancestral allele relative to the derived allele and nucleotide-diversity analysis (*θ*π) to identify very-low-diversity genomic regions. Genes identified by at least two methods were selected. Finally, we excluded genes overlapping regions with a low recombination rate identified by FastEPRR and the result was chosen as the selective-sweep genes respectively for the two buffalo populations (Fig. 3A, Supplementary Table 23 and Supplementary Fig. 29). These analyses identified a total of 801/542 genes bearing selection signatures in the swamp/river-buffalo populations with 67 overlapping genes.

### Selective-sweep genes in swamp buffaloes reflect a domesticated draft-labor role

Recall that domesticated swamp buffaloes perform the majority of draft labor in rice-paddy agro-ecosystems. These animals are exceptionally strong, with pronounced endurance for grueling cultivation-related work. Consistently with their impressive strength and endurance, both KEGG pathway and Gene Ontology (GO) analyses for the selected swamp-buffalo genes revealed enrichment for muscle-related functions and for cardiac-related functions (Fig. 3B and Supplementary Table 24). The most significant KEGG pathway in swamp buffaloes was the ‘Hippo-signaling pathway’ (*P* = 6.10E-04). The Hippo-signaling pathway controls organ size in animals and regulates skeletal and heart-muscle myogenesis/regeneration via interaction with TEAD transcription factors [22]. TEA Domain Transcription Factor 1 (*TEAD1*), as a key transcription factor in the Hippo pathway, had a strong selection signature in swamp buffaloes and was identified by all the tests. Previous studies confirmed that *TEAD1* causes muscle-fiber transition from fast to slow type in mouse, which enhanced the endurance [23,24] (Supplementary Fig. 30). Furthermore, cardiac-related pathways, e.g. ‘Arrhythmogenic right-ventricular cardiomyopathy (ARVC)’ (*P* = 1.00E-03) and ‘negative regulation of cardiac-muscle-cell proliferation’ (*P* = 1.30E-03), also played an important role in adapting the impact of exhausting farming work. For example, five genes (*PRKCI* [25], *LEF1*, *TCF7L2* [26], *CTNNA1*, *CTNNA3* [27]) in the Hippo pathway were essential for the cardiac morphogenesis and functioning. Four genes (*ANK2*, *RYR2*, *CACNA2D1*, *CACNA1C*) associated with the ‘ARVC pathway’ and the ‘regulation of heart rate by cardiac conduction’ were candidates for sudden cardiac death [28]. Future experimental studies aimed at exploring the physiological character of swamp buffaloes are important for improving research on exercise physiology and cardiovascular health to humans.

Another arresting character of swamp buffaloes is their docile and tractable character. In rice-paddy agro-ecosystems, humans trained them for farming rice fields and give starch-rich concentrated feed and soothing as a reward. Our selective-sweep analysis also identified the fascinating trend of substantial enrichment for nerve development and behavior in swamp buffaloes. The KEGG pathways and GO terms enriched for swamp buffaloes included the ‘Oxytocin-signaling pathway’ (*P* = 2.30E-02), ‘Morphine addiction’ (*P* = 2.40E-02), ‘regulation of synaptic transmission, GABAergic’ (*P* = 2.50E-04) and ‘regulation of neuron differentiation’ (*P* = 3.40E-03). The oxytocin-signaling pathway regulated social cognition and behaviors in many psychological and behavioral processes in humans and animals [29,30] and *OXTR* was the key receptor-encoding gene that regulates social cognition and behaviors, including parenting, empathy and using social relationships to manage stress [31]. Additionally, adenylyl cyclase III (*ADYC3*) was involved in all the pathways mentioned above and regulates the transduction of the physical and emotional signals (e.g. favorite food, soothing and care) into cAMP and eventually acting on the brain reward circuits [32] (Supplementary Fig. 31).

Moreover, two critical starch-digestion enzymes, namely *AMY2B* and *SI*, were found in the selective-sweep signals of swamp buffaloes. *AMY2B* encoded a pancreatic α-amylase to catalyse the first step of starch digestion. *SI* encoded a sucrase-isomaltase that is expressed in the intestinal brush border and catalyses the final stage of starch digestion. In the process of training and working, swamp buffaloes were fed with starch-rich concentrated food including rice porridge and sweet potato [5]. The selection of starch-digestion-enzyme genes suggested that swamp buffaloes may have a new mechanism for adapting to the rumen acidosis caused by starch-rich feeding habits unlike other ruminants [33]. One example of starch-rich-diet adaption in the domestication process is represented by dogs, for which an increased copy number of *AMY2B* was identified [34].

### Selective-sweep genes in river buffaloes reflect a domesticated producer role

River buffaloes have many famously prized milk-producing breeds like Nilli-Ravi, Murrah and Italian Mediterranean [6], and they bring the milking performance of a high yield even in hot environments. The most significant KEGG pathway in river buffaloes was the ‘ubiquitin-mediated proteolysis’ (*P* = 6.20E-04) (Fig. 3C, Supplementary Table 25 and Supplementary Fig. 32) and we found that six genes (*HERC1*, *RFWD2*, *SKP1*, *UBR5*, *HERC4*, *CUL5*) had functions regulating the ubiquitination and degradation of the misfolded proteins, which evoke neurotoxicity in many diseases like Alzheimer's and Parkinson's diseases [35]. For example, *HERC1* was the key element in the ubiquitin-proteasome system that was essential for protein degradation [36], which responded to protein aggregation in neurocyte and neurological damage caused by heat-stress conditions [37]. Specifically, there were pathways concerning cardiac functions relating to endurance, which were also observed in swamp buffaloes, e.g. ‘cardiac-muscle contraction’ (*P* = 2.20E-02). It was most likely the traces of selection for river buffaloes as working animals in history.

Essentially, we found many selected sweep genes related to fecundity, milk production and body size (e.g. *IGF2BP2*, *METTL17*, *RNASE2*, *RNASE4*, *ESR1*, *INSR*, *BRCA1*, etc.), which were also reported in the QTL of cattle by other research papers (Supplementary Table 30). For example, *ESR1* is necessary for mediating mammary ductal outgrowth and morphogenesis. *IGF2BP2* [38], *METTL17*, *RNASE2* and *RNASE4* [39] are associated with milk and body-mass traits in cattle, and were displayed with evident selection signals. It would be good practice to combine our results with future studies like GWAS for improving the production performances of river buffaloes.

### Overlapping genes reflect the convergent patterns in the domestication process

Domestication syndrome is a suite of behavioral, physiological and morphological traits in breeding animals not observed in their wild forebears [40,41]. Swamp and river buffaloes were split up more than 1.1 million years ago and independently domesticated. Although their breeding processes were different, domestication syndrome was observed in both buffalo species, including increased docility, reductions in body size, earlier sexual maturity and faster development.

In order to figure out the potential convergent evolution in domestication, we analysed 67 overlapping selective-sweep genes in both buffalo sub-species, which was higher than expected (*P* = 8.97E-13, Fisher's exact test) (Supplementary Tables 26–28 and Supplementary Fig. 33). Those overlapped genes were significantly enriched in two KEGG pathways, namely ‘Long-term depression’ (*P* = 2.20E-03) and ‘hippo-signaling pathway’ (*P* = 4.80E-03), which was consistent with the characteristics for farming work. For instance, bone morphogenetic protein 6 (*BMP6*) is known for its ability to induce cartilage and bone formation [42]; *TCF7L2* and *CTNNA3* are essential for cardiac morphogenesis and functioning. On further examination of the selective-sweep genes in the two buffalo sub-species, as well as in other domesticated animals including dogs, yaks, rabbits, cats and chickens, we found that the selection of neuron-function-related genes was common in these animals (Table 2 and Supplementary Table 29). For example, *GRIK2* and *GRIA2*, encoding two glutamate-receptor proteins and belonging to the mainly excitatory neurotransmitter receptors in the mammalian brain, were reported as selective-sweep genes in most of these species. A previous study revealed that the regulatory changes in glutamate-receptor expression not only attenuate the feedback stress responses, but also lead to improvements in plasticity, learning and cooperation in (self-)domesticated species. Our finding suggests that the selection of a reduced nerve response is the common trait of domestication and is consistent with the hypothesis proposed that domestication syndrome results predominantly from mild neural crest cell deficits during embryonic development [40].

**Table 2.** Description of the behavior-related genes shared by buffaloes and other domesticated animals.

Genes	Performance in functional assay	Other domesticated animals
*GRIK2*	Fear, anxiety and aggression [70]	Rabbit, dog, yak, duck [70]
*GRIA2*	Fear, anxiety and aggression [70]	Cat, pig [70]
*NF1*	Language learning [71]	Chicken [72]
*FOXP2*	Language learning [73]	Dog [74]
*SCN1A*	Epilepsy [75]	Rabbit [76]
*SCN9A*	Pain sensation [75]	Rabbit [76]
*DLG4*	Spatial learning [77]	Cat [78]
*PLCB1*	Schizophrenia [79]	Rabbit [76]
*ALS2*	Motor neuron survive [80]	Chicken [72]
*AMY2B*	Starch digestion	Dog [81]

## DISCUSSION

Our study is the largest buffalo-genome-sequencing project to have been completed to date. We present the first reference genomes for both swamp buffaloes and the Indian river buffaloes, and our sequencing and analysis of 230 geographically disparate individuals comprise the first re-sequencing effort reported for buffalo. Our genomic and large SNP data sets provide long-awaited modern genomic resources for buffalo research. Our study also revealed potential contributions by distinct sets of selected genes in the swamp- and river-buffalo populations that led to divergent behavioral and physiological phenotypes, and to their attendant domesticated agro-ecological niches.

The divergent time between swamp buffaloes and river buffaloes was assessed for the first time using the whole-genome data. According to previous studies by mitochondria, the divergent time is close to 1 million years ago. Our results showed that timing calculated by the distribution of Ks was 1.1 million years ago and timing calculated by MCMCtree was 3.5 million years ago. We also observe incongruity between the mitochondrial and nuclear phylogenies in sub-species of the swamp buffalo. Previous studies [43] proposed two major sub-populations in swamp buffaloes based on the mitochondrial genome, although the whole-nuclear-genome analysis suggests a monomorphic swamp-buffalo population. The phenomenon is also found in other domesticated animals (e.g. horses, dogs and pigs), indicating hybridization in history [44].

Excitingly, the divergence times of swamp and river buffaloes calculated in our analysis coincide with known geological events from the same time period, potentially providing a plausible mechanistic explanation for the forces that drove this divergence. Specifically, the sea-level decline during the Xixiabangma Glacial created a migration corridor for the ancient buffaloes to bypass the India–Myanmar border ranges, thus causing geographical isolation and likely facilitating the fixation of variation in the chromosome numbers and other genetic polymorphisms into the genomes of the two isolated populations. Thus, patterns of buffalo migrations—for example in Laos, Myanmar and Yunnan—may have shaped the apparent hybrid zone from which we sampled individuals harboring a genomic admixture of swamp and river buffaloes. This hypothetical scenario corresponds to our phylogeny, indicating high gene flow amongst buffaloes in certain Southeast Asian regions. This hypothesis about the role of migration patterns in shaping the population genetics of buffaloes could be tested by targeted sampling organized around local topographical features.

Differences in variations and differential selection signatures for genes found in the swamp- versus river-buffalo populations can suggest a new hypothesis driven by functional studies investigating how such differences mechanistically contribute to behavioral and morphological phenotypes for each buffalo sub-species. In light of ongoing agricultural development in China and Southeast Asia, our findings may help hybrid-buffalo breeders to focus their selection targets on the development of buffaloes that retain their docile dispositions and for which they are prized in their farming communities, while maintaining also milk and meat production efficiently. The candidate genes identified here can be used in buffalo-breeding strategies that link swamp buffaloes. For instance, the ‘muscle genes’ like *TEAD1*, the ‘docility genes’ like *OXTR* and *ADCY3*, and the ‘starch-digestion genes’ like *AMY2B* and *SI*, with the ‘milking genes’ like *BRCA1*, *ESR1*, *RNASE2*, *RNASE4* and the ‘growth genes’ like *IGF2BP2* and *INSR*, would represent a precious selection target. Such efforts can go a long way toward overcoming the unstable temperaments and aggressive behaviors that currently limit the successful introduction of milk-producing river and hybrid buffaloes into areas that formerly relied on swamp buffaloes as beasts of burden.

Finally, our study raises some profoundly interesting questions about the nature of the interspecific cooperation that occurs during the domestication and establishment of productive agro-ecosystems. Provocatively, our discovery that genes are shared by domestic animals bearing signatures of recent selection implies the role of potential convergent evolution. However, it is worth mentioning that domestication syndrome is presumed to be a polygenic condition, to which conserved non-coding elements and repeated elements also contribute. Regions with significant selective signals and enrichment of biological pathways and GO terms illustrate that their function is closely related to domestication syndrome. We infer that the identified convergent evolution genes/regions in this unconventional approach were not random or biased by methods. Besides, the enrichment of the ‘Oxytocin-signaling pathway’ and common selection on *OXTR* has been driven likely by the visual and auditory communications that occur daily between humans and buffaloes in rice-paddy agro-ecosystems. Our discovery of cooperative evolution for neurological traits in two interacting species may have revealed plausible gene-level mechanisms that may explain how at least some of these psychological differences were selected by the environment.

## METHODS

### Sample collection and *de novo* sequencing

All animal work was approved by the Guangxi University Institutional Animal Care and Use Committee. For the reference-genome sequencing, one female Fuzhong swamp buffalo and one female Murrah river buffalo were sampled (DNA from blood) from the Buffalo Research Institute of the Chinese Academy of Agricultural Sciences and Guangxi Zhuang Autonomous Region. For context, the Fuzhong swamp buffalo is one of the most famous local breeds in the Guangxi Zhuang Autonomous Region of China and the river buffalo was the offspring of the Murrah buffalo that was officially introduced from India by the Chinese government in 1957. *De novo* genome sequencing was performed on these two buffaloes: for the swamp buffalo, we generated 152-Gb (57.8-fold) PacBio (RSII) single-molecule long reads (average read length of 8.6 kb) and 179-Gb (67.8-fold) pair-end sequencing (Illumina HiSeq, PE250); for the river buffalo, we generated 47-Gb (17.8-fold) PacBio single-molecule long reads (average read length of 13 kb) and 181-Gb (68.4-fold) of pair-end sequencing (Supplementary Table 2). The genome sizes, as estimated by kmer analysis, were 2.65 GB for both buffalo sub-species (Supplementary Table 4).

### *De novo* assembly

A PacBio-only assembly was performed using Wtdbg (https://github.com/ruanjue/wtdbg-1.28), which assembles raw reads (without error correction) and then builds the consensus from intermediate assembly output. To significantly reduce the remaining INDEL and base-substitution errors in the draft assembly, Quiver [45]—a quality-aware consensus algorithm applying the rich quality scores embedded in Pacific Biosciences' bas.h5 files—was initially used. Pilon [46] was used to improve the local base accuracy of the contigs via analysis of the read-alignment information based on paired-end bam files (twice). The initial assembly had N50 sizes of 8.8 and 3.1 Mb for the swamp-buffalo and river-buffalo reference genomes, respectively. About 135-fold coverage optical maps for both swamp buffaloes and river buffaloes were produced with molecules N50 252 kb. Bionano's hybrid scaffolding pipeline [47] was used to combine the assembly and putative nicking endonuclease-specific recognition sites in the reference into hybrid scaffolds and the resulting scaffolds were then clustered using high-throughput chromosome conformation capture (Hi-C) [48] into chromosome-scale scaffolds (Supplementary Tables 5 and 6).

### Assessment of the genome quality

Four methods were used to evaluate the quality of the genomes. First, separately from the PacBio-only assembly, we also *de novo* assembled the reference genomes using Illumina paired-end reads (PE250) by SOAPdenovo2 [49]. We compared the PacBio-only assemblies and the Illumina-only assemblies using QUAST [50] and focused on the result of mismatches per 100 kb. We assumed that the mismatches between the two assemblies represented incorrect nucleotides and heterozygotes. Second, all of the Illumina paired-end reads were mapped to the final genome using BWA [51] and SNPs were called using Samtools [52]. The predicted error rate was calculated using the homozygous substitutions divided by the length of the whole genome, which included the discrepancy between assembly and sequencing data. Third, we used the RNA-sequencing strategy for swamp buffaloes detailed in Supplementary Table 3 and the RNA of river buffaloes were downloaded in NCBI (BioProject number: PRJEB4351). We then used the RNA-seq data for a *de novo* assembly using Trinity [53]. The assemblies were mapped to the swamp-buffalo reference genome and we assessed the mapping rates and coverage. Finally, we checked core gene statistics using BUSCO [54].

### Synteny analysis of chromosomes

To identify the chromosomal rearrangements among cattle, swamp buffaloes and river buffaloes, alignments between the two buffalo sub-species and between buffaloes and cattle were performed using the nucmer program in the mummer package [55]. Homologous-synteny block maps were plotted using Perl script.

### Gene-family expansion and contraction

In addition to the genome data that we generated for the two buffalo sub-species, we downloaded genome-annotation information for humans, cattle, yaks, mice, dogs, horses, sheep and goats in ensemble (http://ensemble.org, release-87). We chose the longest transcript to represent each gene and removed genes with open reading frames shorter than 150 bp. Gene-family clustering was then performed using OrthoMCL [56,57] based on the predicted gene set for 10 genomes. This analysis yielded 17 890 gene families. To identify gene families that had undergone expansion or contraction, we applied the CAFE [58] program, which employs a probabilistic graphical model to infer the rate and direction of the change(s) in gene-family size over a given phylogeny. Functional-enrichment analysis of GO terms and pathways was performed using the functional-annotation tools from InterPro and KEGG.

### Phylogeny analysis and positively selected genes

We used two methods to evaluate the divergent time between swamp buffaloes and river buffaloes. (1) We used orthologs of swamp buffaloes, river buffaloes, cattle and goats from the 11 628 single-copy gene clusters determined by the OrthoMCL. The synonymous substitution rates (Ks) of all pairwise combinations were estimated using the yn00 program in PAML. The peak of the Ks bin was used to estimate the divergence time between different genomes (Supplementary Fig. 13). (2) We constructed a phylogenetic tree based on 4-fold degenerate third-codon transversion (4dtv) in the sequence alignment of 6429 single-copy gene families from swamp buffaloes, river buffaloes and the 8 other mammals with the GTRGAMMA model. Divergence times were estimated using PAML [59] MCMCTREE. A Markov chain Monte Carlo process was run for 2 000 000 iterations, with a sample frequency of 100 after a burn-in of 1000 iterations under an independent-rates model. Two independent runs were performed to check convergence. The following constraints were used for time calibrations: (i) the divergence time between B*ovinae* and *Caprinae*: 18–22 Ma; (ii) the divergence time between *Ruminantia* and *Suina*: 48.3–53.5 Ma; (iii) the divergence time between *Euarchontoglires* and *Laurasiatheria*: 95.3–113 Ma [60]. Because molecular-clock interpolation using divergent times of higher taxa could yield overestimates, we used the divergent time between swamp buffaloes and river buffaloes estimated by Ks distribution in the end. Based on multiple sequence alignments, positive selection of genes in the swamp- and river-buffalo reference genomes were tested under the phylogenetic tree. We used the branch-site model, which allowed ω to vary both among sites in the protein and across branches on the tree, and which aims to detect positive selection affecting a few sites along particular lineages. Comparison between Model A and null Model B was performed with likelihood-ratio tests (LRTs) implemented in the Codeml program in PAML. Significance (*P* < 0.05) of the compared LRTs was calculated via chi-square tests, assuming that the null distribution was a 50:50 mixture distribution with a point mass at zero.

### Estimating effective population size in history

The demographic histories of four taxa in the Bovinae, including the two aforementioned sub-species of Bubalus, one species of Syncerus (the wild African buffalo) and one species of Bos (cattle), were inferred using a hidden-Markov-model approach as implemented via PSMC [61] based on the SNP distribution (Fig. 1C). We transferred the BAM file to a whole-genome diploid consensus sequence and the parameters used in the program ‘psmc’ were set as follows: −N30 −t15 −r5 −p ‘4+25 * 2+4+6’ to infer the population-size history. Long chromosomes were divided into shorter segments to perform 100 rounds of bootstrapping. The generation time (g) of swamp buffaloes, river buffaloes and African buffaloes was set to 6 years, while that of cattle was set to 5 years. The neutral mutation rate per year (μ/y) used was 2.2 × 10^−9^ for all four taxa. This was selected based on reported research about mutation rates in mammalian genomes [62].

### Re-sequencing samples

We also conducted a re-sequencing analysis in which we sampled 132 swamp-buffalo (2 N = 48) individuals from 7 countries (China-CN, Vietnam-VN, Thailand-TH, Laos-LA, Myanmar-MM, Indonesia-ID and Philippines-PH) and 98 river-buffalo (2 N = 50) individuals from 7 countries (China-CN, Pakistan-PK, Nepal-NP, Iran-IR, Iraq-IQ, Bengal-BD and Italy-IT). The classification of the buffaloes into one or the other sub-species was based on their geographical location. Total genomic DNA was extracted from the blood of the buffaloes using a standard phenol-chloroform protocol. For each individual, at least 5 μg of genomic DNA was used to construct paired-end libraries with an insert size of 300 bp. Sequencing was performed on the Illumina HiSeq 2000 platform (the read length was PE150).

### SNVs calling

All sample data were mapped to both buffalo-genome assemblies simultaneously using BWA-MEM, with default parameters [63]. The delivered mapping reads were then transferred to BAM and sorted using Samtools [52]. In order to reduce the number of miscalls of INDELs in the BAM, we realigned the raw gapped alignment with the Broad's GATK Realigner [64]. This requires two steps: (i) GATK’s RealignerTargetCreator was used to identify suspicious intervals, which need realignment to generate a list of INDELs for every sample; (ii) GATK’s IndelRealigner was run to the realigner over these intervals and to output the realigned BAM file. After alignment, we performed SNP calling on a population scale for the swamp and river buffaloes using a Bayesian approach as implemented in Samtools [52]. The ‘mpileup’ command was used to identify SNPs with the parameters as ‘–redo-BAQ –count-orphans -t -uf’. We also used ‘the bcftools call’ command in Bcftools [65] in conjunction with Samtools mpileup and the parameters were ‘−m −v −O u −’. We retained the high-quality SNPs with coverage depth ≥2 and minimum of two non-reference bases for variants. Because of the relative low sequencing coverage depth of the re-sequencing samples, an MAF method was applied for filtering SNPs: we retained sites with a MAF >2.5%. This pipeline produced several SNV data sets to conduct follow-up analysis. In the phylogenetic and population genetic analyses and Fst calculation, we used the genotypes of all samples with a swamp-buffalo reference genome. In the genome-wide selective-sweep scans and diversity analysis, we used the data sets that contained SNP generated by (i) all swamp buffaloes mapping to the swamp-buffalo reference and (ii) all river buffaloes mapping to the river-buffalo reference.

### Phylogenetic and population genetic analyses

Combining the genotype of cattle and African buffaloes with the SNP data set that we had produced, an NJ tree was constructed with PHYLIP v3.697 (http://evolution.genetics.washington.edu/phylip.html) with a matrix of pairwise genetic distances (cattle sequencing data: GeneBank accession number SRX994908 and African-buffalo sequencing data: Genebank accession number SRX2069824). It should be noted that, although we did not use SNPs for sex chromosomes in any of the analyses presented in the main figures of the study, the SNPs from the Y chromosomes of the re-sequencing individuals were identified based on mapping sequence data to the cattle genome (Btau_5.0.1). FigTree (http://tree.bio.ed.ac.uk/software/figtree/) was used to visualize the phylogenetic trees (Supplementary Figs 19–21). PCA of the SNPs was performed using the smartpca program in EIGENSOFT v5.0 [66]. ADMIXTURE [67] was used to estimate the admixture proportions (from K = 2 to K = 10) with a maximum-likelihood approach and the EM algorithm. Genetic diversity (*θ*_π_) for the buffalo populations (clustered by sampling country) was calculated in 100-kb sliding windows using VCFtools [68].

### Genome-wide selective-sweep scans

To identify genomic regions harboring artificial selection signatures in swamp buffaloes and river buffaloes, we used (i) Sweepfinder2 to scan for selective sweeps using the spatial distribution of allele frequencies (window size: 1000 bp, cut-off = 1%), (ii) the integrated haplotype score method (iHS) to measure EHH at a given SNP along the ancestral allele relative to the derived allele (window size = 100 Kb, cut-off = 5%, which the lowest iHS value was 0.1165 for swamp buffalo and 0.1209 for river buffalo) and (iii) nucleotide-diversity analysis (θπ) to identify very-low-diversity genomic regions (window size = 50 Kb, cut-off = 5%, which the highest θπ in windows was 5.74 × 10^−4^ for swamp buffaloes and 7.99 × 10^−4^ for river buffaloes). Genes identified by at least two methods were selected. Ultimately, we excluded genes overlapping regions with a low recombination rate identified by FastEPRR and the result was chosen as the selective-sweep genes respectively for two buffalo populations. We subsequently used DAVID 6.8 [69] (https://david.ncifcrf.gov/) to assess enrichment of these selected gene clusters. To further research domesticated genes, the *θ*_π_, *θ*_π_ (swamp buffalo)/*θ*_π_ (river buffalo), Tajima’ *D* and *F*_st_ value on a per-site basis and in the 10-kb sliding windows were calculated. We compared the value of divergent genes, sweep-selective genes and all genes, and drew a box plot (Supplementary Fig. 29).

## DATA AVAILABILITY

The whole-genome sequence data reported in this paper have been deposited in the Genome Warehouse in BIG Data Center75, Beijing Institute of Genomics (BIG), Chinese Academy of Sciences, under accession number GWHAAJZ00000000 and GWHAAKA00000000 that are publicly accessible at http://bigd.big.ac.cn/gwh. The genome re-sequencing reads for two buffalo sub-species have been deposited into the genome sequence archive (GSA) in BIG under accession code CRA001463. The RNA-sequencing reads for multiple tissues of swamp buffaloes have been deposited into the GSA in BIG under accession code CRA002325. The variation data reported in this paper have been deposited in the Genome Variation Map (GVM)76 in BIG under accession numbers GVM000043 that are publicly accessible at http://bigd.big.ac.cn/gvm/getProjectDetail?project=GVM000043.

## Supplementary Material

nwaa024_Supplemental_Files
