# Supplementary material for: Understanding divergent domestication traits from the whole-genome sequencing of swamp- and river-buffalo populations
Source: Natl Sci Rev. 2020 Feb 17;7(3):686–701. doi: 10.1093/nsr/nwaa024 (PMC8289072; doi:10.1093/nsr/nwaa024)
Supplement: nwaa024_Supplemental_Files [file nwaa024_supplemental_files.zip › SUPPLEMENTARY_MATERIALS_12-Feb-2020_ AP revision.docx]

**SUPPLEMENTARY MATERIALS**

**Supplementary Text**

The hypothesis about buffalo population divergence caused by sea level change in Pleistocene glacial

Combining the result of PSMC analysis and previous studies about Pleistocene glacial [1-3], we found that buffalo historical population size fluctuations happen to dovetail with the sea level change. During the Xixiabangma Glacial (0.80~1.17Ma) the sea level dropping made continent of Sundaland (consist of modern insular Indonesia and Malaysia) appeared. The continental shelf corridor from Sundaland to South Asia had formed for the first time. The land exposed after the seawater receded was initially covered with herbaceous plants to form a savanna corridor. The river buffalo ancestors migrated through this savanna corridor to the plains of South Asia. During the subsequent interglaciation (0.72~0.80Ma), the sea level raised and re-drown the corridor, which made isolation of the two buffalo populations. For the same reason, in the penultimate interglaciation (0.30~0.50Ma), the divergence of the two buffalo populations became more obvious. In the more severe Penultimate Glaciation (0.13~0.30Ma), the time of sea level declines was longer and the continental shelf corridor from Sundaland to South Asia had appeared again. On the one hand, hybridization events may have occurred when buffaloes from Sundaland migrated to South Asia, thus made the sharply rising population curve of river buffalo. On the other hand, the habitat area expansion may have contributed to the population expansion.

The expanded *Hsp90A* and *ABCC4* genes

Another significantly expanded gene was *ABCC4*, known as the multidrug resistance-associated protein 4 (*MRP4*), which is a member of multidrug resistance protein (MRP) family acting as efflux pumps relevant to drugs distribution and toxins detoxification[4]. Water buffaloes eat a wide range of plants, including many herbs growing everywhere in the jungle. Many of these plants can be used as medicine to cure infections but also may be fatal if consumed in great quantities. *MRP4* transporters located on the apical membrane of nephric tubules facilitate efflux of these toxins to urine[5], thereby reducing the impact of the toxins.

Heat shock protein *Hsp90AA1* subfamily expanded in water buffalo. It encodes heat shock protein 90α (*Hsp90α*), a stress-inducible molecular chaperone, which is related to the adaptation to environmental stress. Due to the lack of sweat glands, water buffaloes have a poor ability of thermoregulation, thus they easily suffer hyperthermia under working and torridity circumstances. The expansion of *Hsp90AA1* potentially increased these molecular chaperones abundance thereby might reduce the damage of hyperthermia. Hsp90 also mediates as bacterial lipopolysaccharide (LPS) activators that stimulating inflammatory response to defence bacterial infection[6].

Summarized pipeline

1. Pipeline of De novo assembly

(1) Wtdbg 1.2.8:

wtdbg-1.2.8 -i pacbio.fa.gz -k 0 -p 17 -K 0.05 -S 4 -fo dcg --rescue-low-cov-edges --tidy-reads 5000 --edge-min 2

(2) SOAPdenovo2:

SOAPdenovo-63mer all -s config.txt -o out/out -K -p -R

(3) Bionano irys 2.4:

python pipelineCL.py -U -d -T 20 -j 16 -N 2 -i 5 -a "buffalo.xml" -w -t "latest" -l "output" -b "Molecules.bnx" -C "clusterArguments.xml"

hybridScaffold.pl -f -B 2 -N 2 -r RefAligner -n contig.fa -b maps.cmap -c hybridScaffold_config.xml -o scaffold -y -m RawMolecules.bnx

(4) Hi-C:

bwa aln chromosome.fa left.fq > left.sai

bwa aln chromosome.fa right.fq > right.sai

bwa sampe -f pair-end.sam chromosome.fa left.sai right.sai left.fastq right.fastq

PreprocessSAMs.sh

Lachesis <your-INI-file>

CreateScaffoldedFasta.pl

2. Pipeline of genome quality assessment

(1) QUAST 5.0.0:

quast.py illumina_assembly.fa -r chromosome.fa -o output

(2) BWA 0.7.5a-r405 + Samtools 1.6

bwa mem -t 10 -M -R "@RG\tID:<ID>\tLB:<LIBRARY_NAME>\tSM:name\tPL:ILLUMINA" chromosome.fa left.fq right.fq | samtools view -bS -h - | samtools sort -@ 10 -o sort.bam

samtools mpileup --redo-BAQ --count-orphans -t DP -uf chromosome.fa -b sort.bam | bcftools call -m -v -O u - > vcf

(3) Trinity 2.1.1 mapping:

Trinity --left rna1.fq --right rna2.fq --seqType fq --SS_lib_type RF --CPU 5 --jaccard_clip --normalize_reads --output denovo --bflyCalculateCPU

blat chrmosome.fa Trinity.fa -q=dna output.psl

(4) BUSCO :

Run_BUSCO.py -i chromosome.fa -l embryophyta_odb9 -o busco_out -m genome --cpu 10

3. Pipeline of Synteny analysis

(1) Mummer4:

nucmer -t –p out chromosome.fa query.fa

delta-filter -m out.delta

show-diff out.delt

(2) LASTZ:

lastz =254 K=4500 L=3000 Y=15000 T=2 --format=lav –output

lavToAxt –fa stdout | axtChain -linearGap=medium stdin stdout | chainPreNet stdin

chainNet

netSyntenic

netToAxt stdout | axtSort stdin stdout | axtToMaf stdin

4. Annotation pipeline

(1) RepeatModeler 1.0.11:

BuildDatabase -name buffalo -engine ncbi chromosome.fa

RepeatModeler -engine ncbi -pa 5 -database buffalo

Repeatmask 4.0.9:

RepeatMasker -pa 16 -dir ./result -s -xsmall -gff -species Ruminant chromosome.fa

(2) De novo prediction：

Augustus 3.03:

augustus --gff3=on --noInFrameStop=true --softmasking=1 --species=human chromosome_mask.fa >augustus.gff

(3) Glimmer HMM 3.0.2:

glimmerhmm_linux_x86_64 genome.hardmask -d GlimmerHMM/trained_dir/human -n 1 -g >glimmerhmm.gff

(4) Genscan:

snap snap/HMM/mam39.hmm genome.hardmask -gff -quiet >snap.gff

(5) SNAP:

genscan Genscan/HumanIso.smat genome.hardmask > genscan.gff

(6) GeneWise 2.4.1:

Genewise protein.fa dna.fa -both -gff

(7) Trinity 2.1.1:

Trinity --left rna1.fq --right rna2.fq --seqType fq --SS_lib_type RF --CPU 5 --jaccard_clip --normalize_reads --output denovo --bflyCalculateCPU

(8) TopHat2:

Botwtie2-build chromosme.fa chromosome

tophat -N 3 --reads-edit-dist 3 -p 4 -i 20 -I 4000 --mini-segment-intron 20 --max-segment-intron 4000 --min-coverage-intron 20 --max-coverage-intron 4000 --coverage-search --microexon-search -G genome.gtf -o tophat chromosome tophat1.fq tophat2.fq

(9) EvidenceModeler (EVM):

evidence_modeler.pl -G chromosome.fa -g denovo.gff3 -w weights.txt -e transcript.gff -p homolog.gff -r chromosome.out.gff --exec_dir out

5. Pipeline of gene family expansion and contraction

(1) Orthomcl 2.0.9:

orthomclInstallSchema orthomcl.config install_schema.log

orthomclBlastParser all_VS_all.out.tab compliantFasta 1>similarSequences.txt 2>err.log

orthomclLoadBlast orthomcl.config similarSequences.txt

orthomclPairs orthomcl.config orthomcl_pairs.log cleanup=no

orthomclDumpPairsFiles orthomcl.config

mcl mclInput --abc -I 1.5 -o mclOutput

orthomclMclToGroups GF 1 < mclOutput > groups.txt

6. Phylogeny analysis and identification of positively selected genes

(1) Gblocks 0.91b:

Gblocks protein.fa -t=p

RAxML 8.1.24:

raxmlHPC -PTHREADS-SSE3 -f a -x 12345 -p 12345 -# 100 -m PROTGAMMAILGX -s ex.phy -T 5

(2) PAML4.8:

mcmctree

codeml

yn00

7. Estimating the demographic history with effective population size analysis

PSMC 0.6.5-r67:

samtools mpileup -C50 -uf chromosome.fa bam | bcftools view -c - | vcfutils.pl vcf2fq -d 10 -D 100 | gzip > diploid.fq.gz

fq2psmcfa -q20 diploid.fq.gz > diploid.psmcfa

psmc -N25 -t15 -r5 -p "4+25*2+4+6" -o diploid.psmc diploid.psmcfa

psmc2history.pl diploid.psmc | utils/history2ms.pl > ms-cmd.sh

psmc_plot.pl diploid diploid.psmc

8. Pipeline of SNP calling

bwa mem -t 10 -M -R "@RG\tID:<ID>\tLB:<LIBRARY_NAME>\tSM:sample\tPL:ILLUMINA" chromosome.fa sample1.fq.gz sample2.fq.gz | samtools view -bS -h -| samtools sort -@ 10 -o sample.bam

samtools index sample.bam

java -jar GenomeAnalysisTK.jar -R chromosome.fa -T RealignerTargetCreator -I sample.bam -o sample.intervals

java -jar GenomeAnalysisTK.jar -R chromosome.fa -T IndelRealigner -targetIntervals sample.intervals -o sample.realign.bam -I sample.bam

samtools index sample.realign.bam

java -jar SortSam.jar INPUT=sample.realign.bam OUTPUT=sample.realign.sort SORT_ORDER=coordinate

java -Xmx2g -jar MarkDuplicates.jar VALIDATION_STRINGENCY=LENIENT INPUT=sample.realign.sort OUTPUT=sample.realign.sort.bam METRICS_FILE=sample.METRICS

samtools index sample.realign.sort.bam

samtools mpileup --redo-BAQ --count-orphans -t DP -uf chromosome.fa -b bam.list | bcftools call -m -v -O u - > samples.bcf

bcftools view samples.bcf > samples.vcf

9. Genome-wide selective sweep analysis

(1) Sweepfinder2:

SweepFinder2 –f CombinedFreqFile SpectFile

SweepFinder2 -lg 1000 FreqFile SpectFile outfile

(2) Beagle 4.1:

java -Xmx8g -jar beagle.21Jan17.6cc.jar gt=samples.vcf nthreads=14 out=samples_beagle excludesamples=exclude.list

java -Xmx8g -jar beagle.21Jan17.6cc.jar gt=samples.vcf ref=ref.vcf impute=false nthreads=15 out=samples_phase excludesamples=exclude.list

(3) selscan 1.2.0a:

selscan --ihs --vcf samples_beagle.vcf --map samples.map --out samples.ihs

(4) Vcftools 0.1.13:

vcftools --gzvcf samples.vcf.gz --keep samples.list --window-pi 50000 --window-pi-step 25000 --out samples.pi

**REFERENCES**

1. H. K. Voris, Maps of Pleistocene sea levels in Southeast Asia: shorelines, river systems and time durations. *Journal of Biogeography* **27**, 1153 (2000).

2. M. I. Bird, D. Taylor, C. Hunt, Palaeoenvironments of insular Southeast Asia during the Last Glacial Period: a savanna corridor in Sundaland? *Quaternary Science Reviews* **24**, 2228 (2005).

3. C. Mei *et al.*, *Genetic Architecture and Selection of Chinese Cattle Revealed by Whole Genome Resequencing*. (2017), pp. msx322.

4. D. Schrenk *et al.*, Up-regulation of transporters of the MRP family by drugs and toxins. *Toxicology Letters* **120**, 51 (2001/03/31/, 2001).

5. J. J. Kohler *et al.*, Tenofovir renal proximal tubular toxicity is regulated By OAT1 and MRP4 transporters. *Laboratory Investigation* **91**, 852 (03/14/online, 2011).

6. K. Triantafilou, M. Triantafilou, R. L. Dedrick, A CD14-independent LPS receptor cluster. *Nature Immunology* **2**, 338 (04/01/online, 2001).

**SUPPLEMENTARY FIGURES**


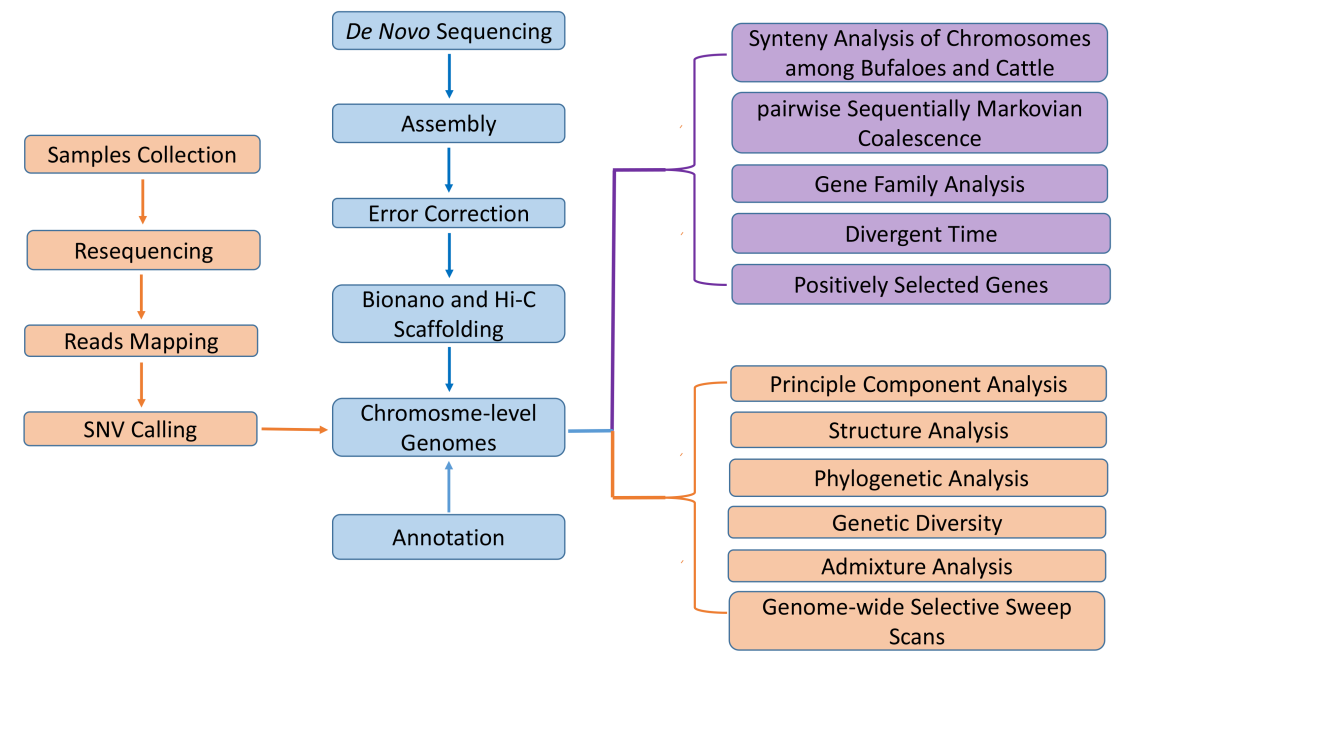


### Supplementary Fig. 1

### Overview of the processing pipeline used for the assembly of the two sub-species of water buffalo and the analysis of the genomes and resequencing data.


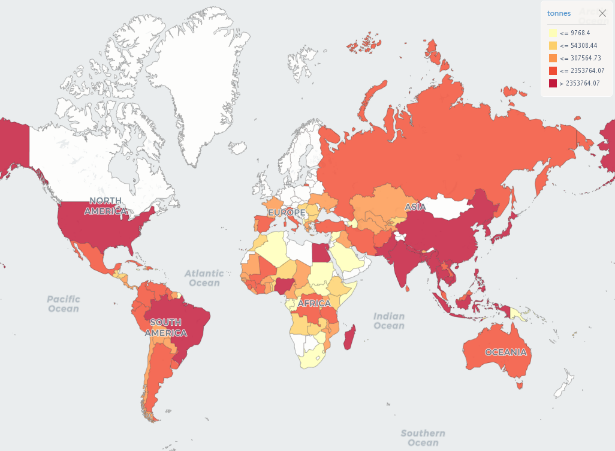

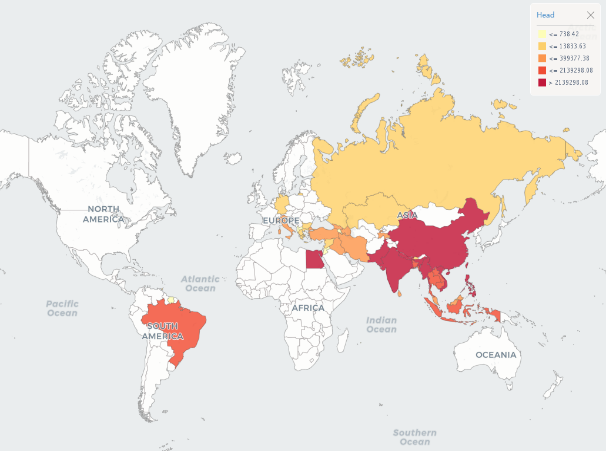


**Supplementary Fig. 2**

The geographical distribution of rice and water buffalo production. (A) Average rice production by country in 1994~2017. (B) Average water buffalo living herds production by country in 1994~2017. Data source from the UN FAO.

|  | Fuzhong buffalo | Murrah buffalo |
| --- | --- | --- |
| Appearance | 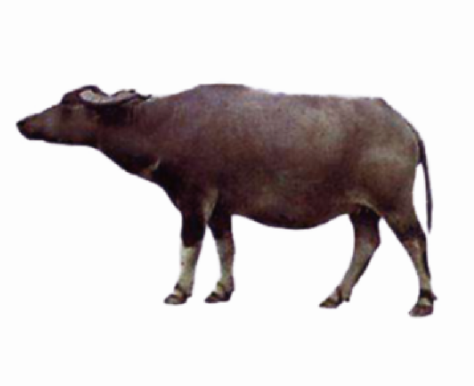 | **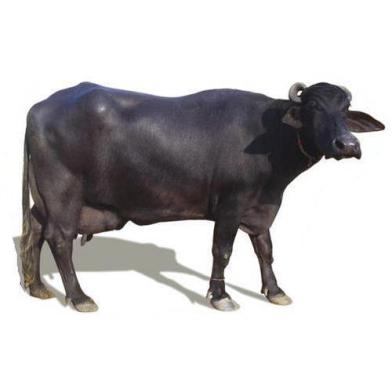** |
| Introduction | Fuzhong buffalo is a mountain swamp buffalo breed raised in Guangxi Zhuang Autonomous Region of China and maintained for draught purposes. It has the advantages of strength, endurance, docile, rough feeding and diseases resistance. | Murrah buffalo is a famous dairy river buffalo breed originally from Punjab and Haryana states of India. It is the most diffuse breed in the world. China imported Murrah buffalo from India in 1957, mainly raised in Guangxi Zhuang Autonomous Region for dairy production and buffalo hybridization breeding. |
| Characteristics | - Grey skin. - Large body size. The average body weight: male 510 is kg, female is 450 kg. - Horns grow outward and curve in a semicircle: male’s horns are shorter but stouter like shark fins; female’s horns are thinner but much longer like sickles. - Stout built and muscularity. They have strong strength and extremely endurance to make them work for a long time in muddy rice paddy. - Docile and easily trained. | - Black skin. - Large body size. The average body weight: male is 750 kg, female is 650 kg. - Horns are short and tightly curled. - Excellent dairy performance. Average milk yield is 1800 kg per lactation duration. Milk fat percentage reached 7.2%. - Usually they are placid, but some individuals are nervous aggressive in front of strangers. |

### Supplementary Fig. 3

### Comparison of swamp buffalo Fuzhong breed and river buffalo Murrah breed.

###
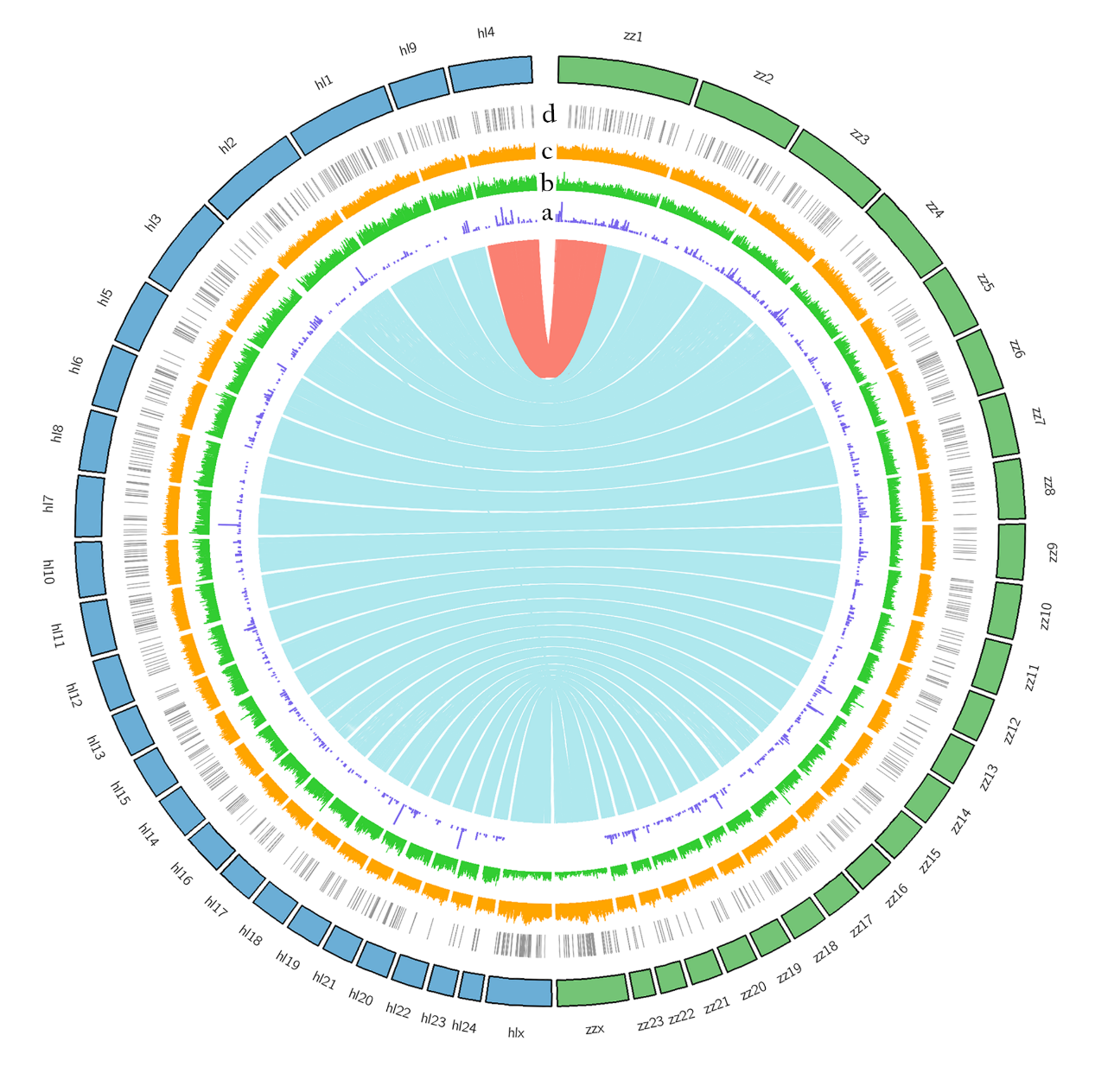


**Supplementary Fig. 4**

Circos plot of the swamp buffalo and river buffalo. The green diagrams outside represent the chromosomes of swamp buffalo (zz) and blue for river buffalo (hl). The links inside represent the synteny between two sub-species of buffalo and the red colour mean reverse direction. a. Distribution of selective sweep genes in the chromosomes. b. Distribution of genetic diversity θ (4 Nu) in 1Mb non-overlapping windows. c. Distribution of genetic differentiation (FST) in 1Mb non-overlapping windows. d. Segmental duplications (length >8 kb).


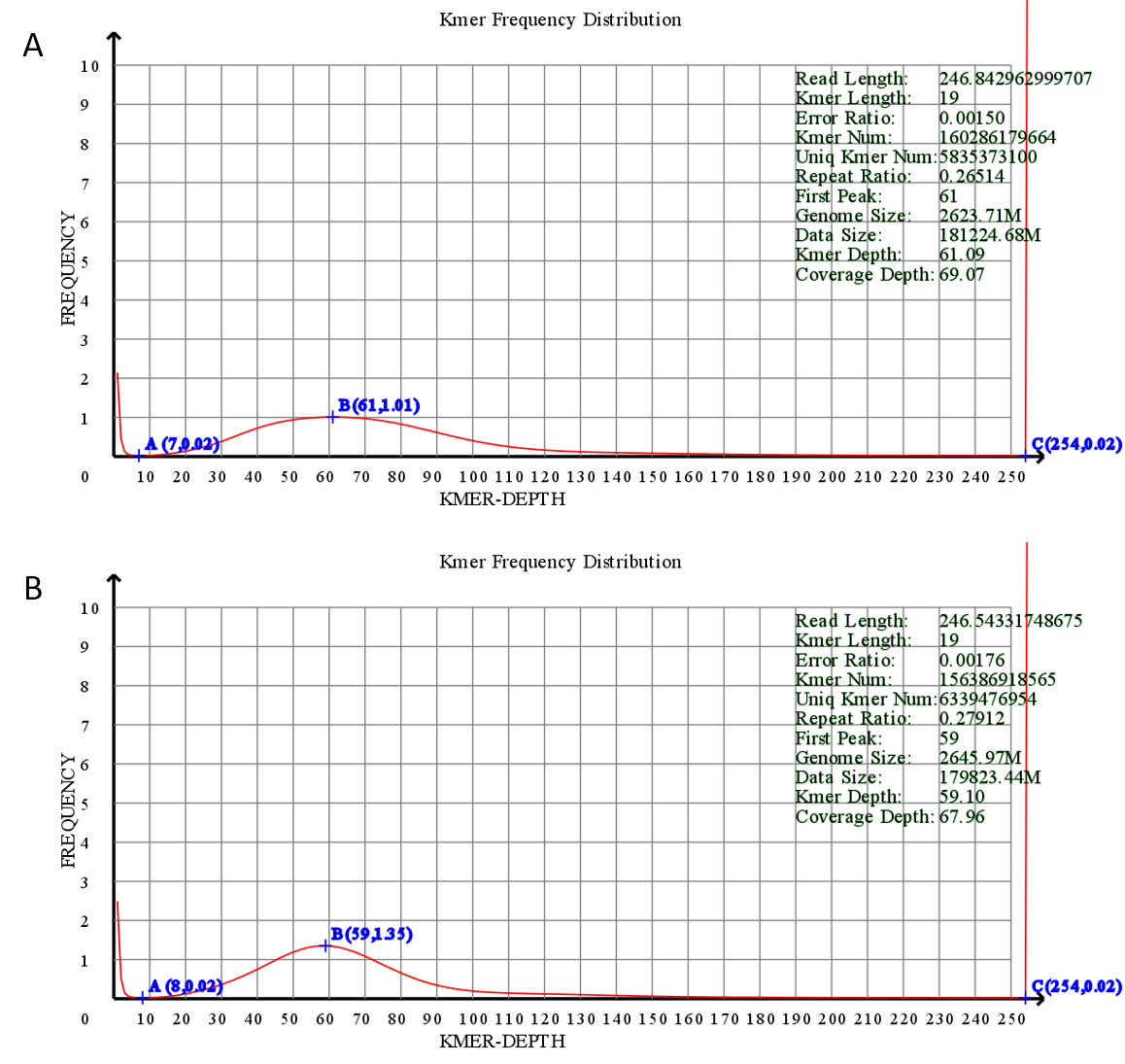


**Supplementary Fig. 5**

Distribution of 19-mer frequency for (A) swamp buffalo and (B) river buffalo. In total 181.2 and 179.8 Gb of high-quality short-insert reads (250 bp) were used to generate the 19-mer depth distribution curve frequency information for two sub-species of buffalo.


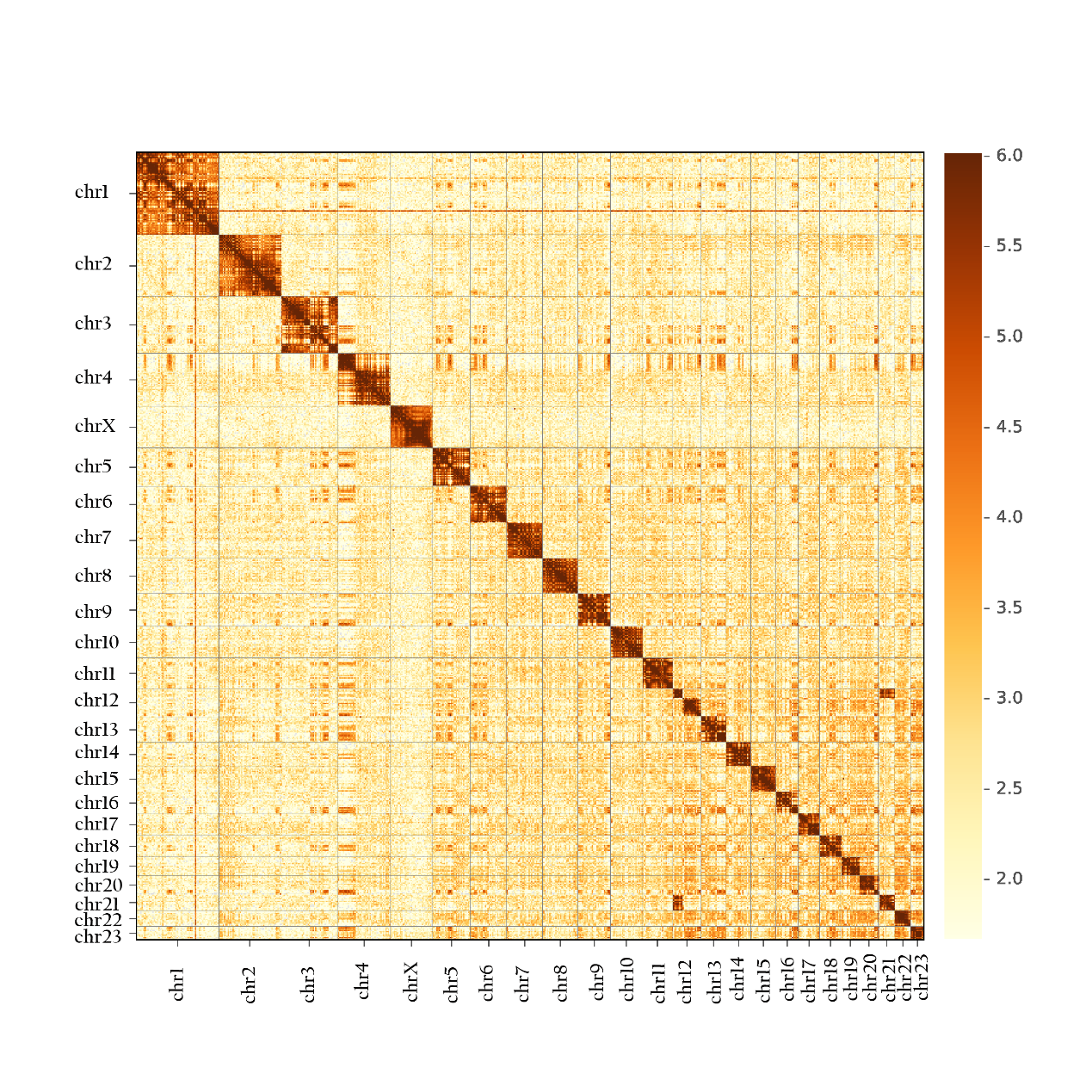


**Supplementary Fig. 6**

Genome-wide all-by-all Hi-C interaction in swamp buffalo.


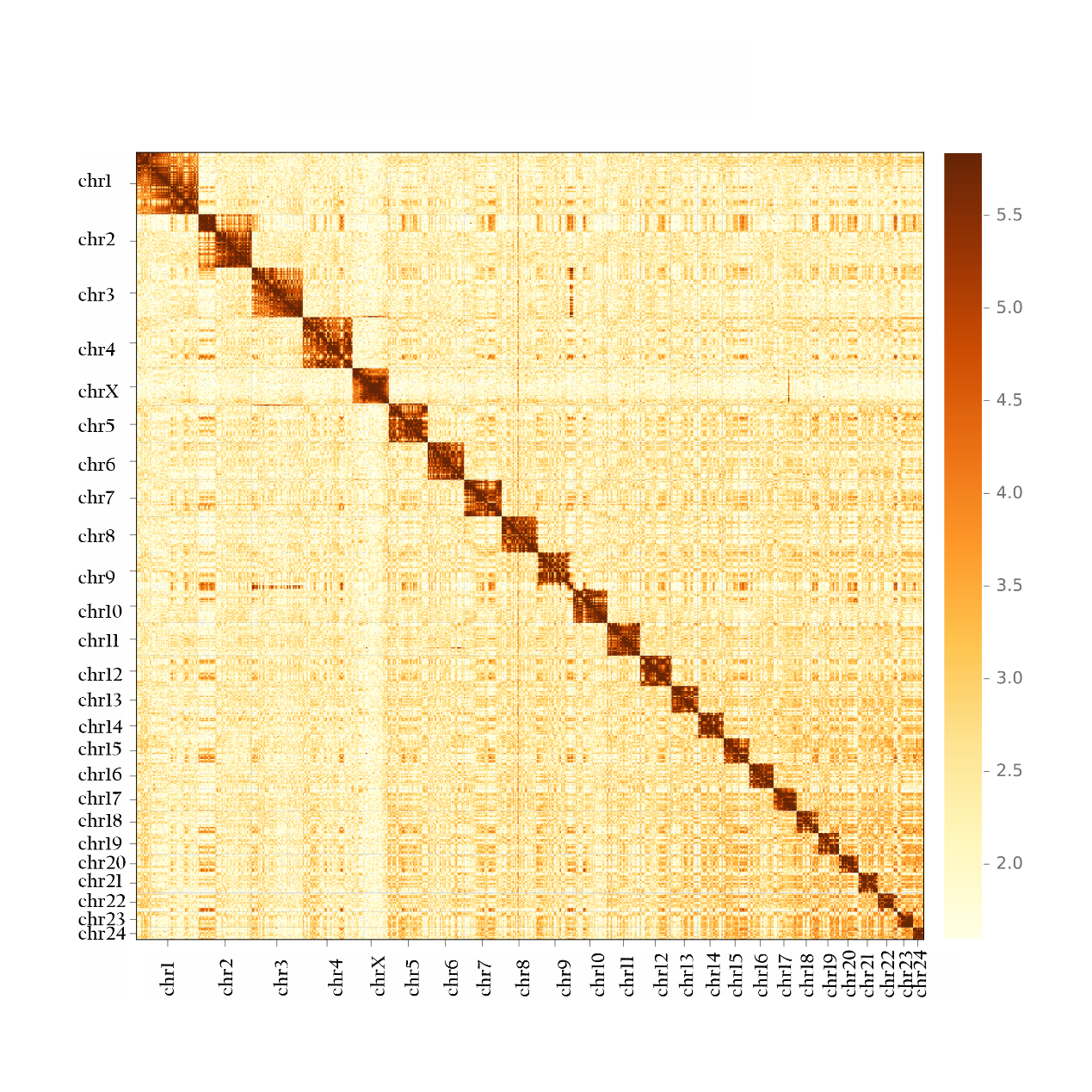


**Supplementary Fig. 7**

Genome-wide all-by-all Hi-C interaction in river buffalo.


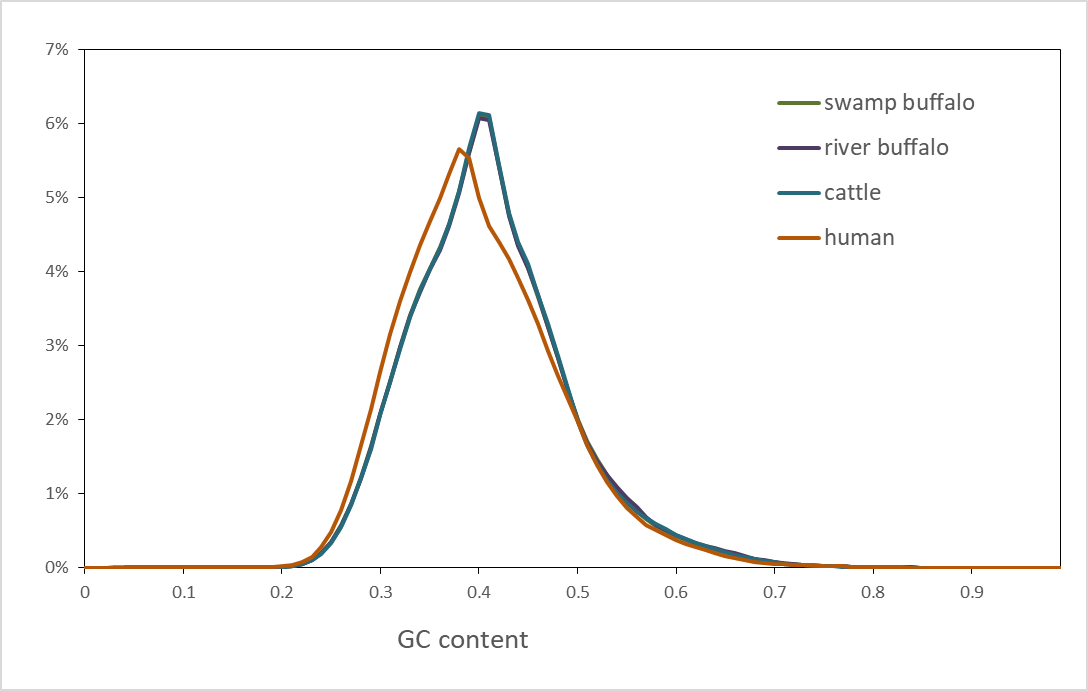


**Supplementary Fig. 8**

Distribution of GC content in swamp buffalo (green), river swamp (purple), cattle (blue) and human (orange) genomes. The proportion of 500 bp non-overlapping sliding windows with a given GC content is shown.


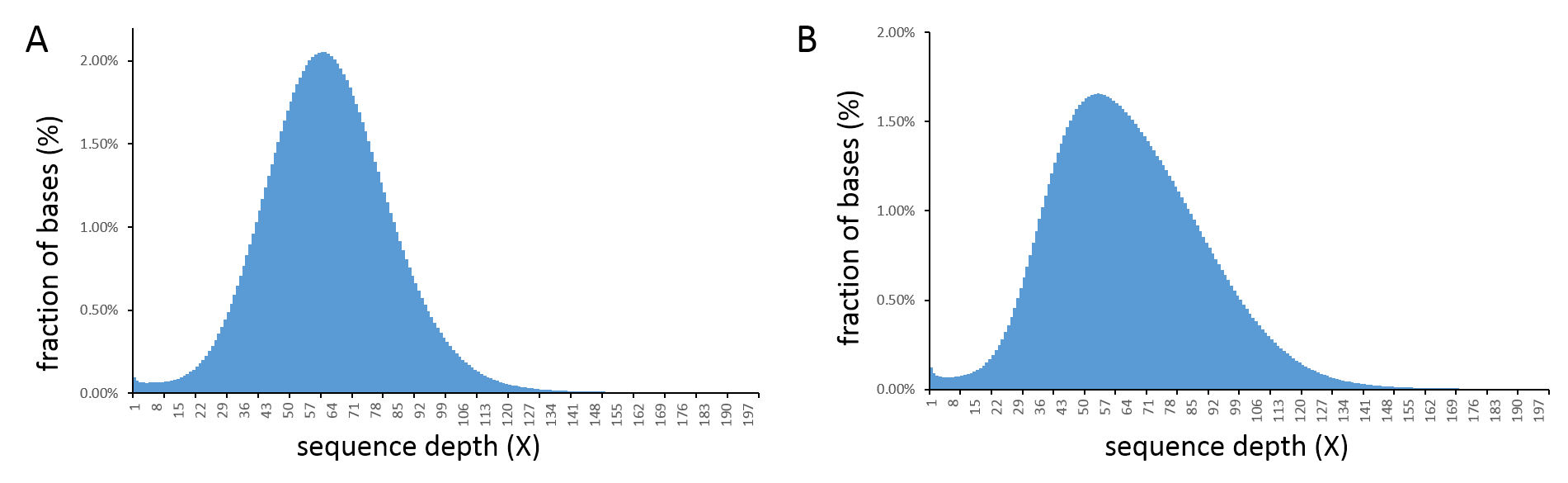


**Supplementary Fig. 9**

Depth distribution of fraction bases for (A) swamp buffalo and (B) river buffalo. The x-axis represents the sequencing depth, and the y-axis the fraction of bases. The high-quality short-insert reads (250 bp) were mapped to the swamp buffalo genome assembly with an average depth of 64.0 and 65.4 for swamp buffalo and river buffalo.


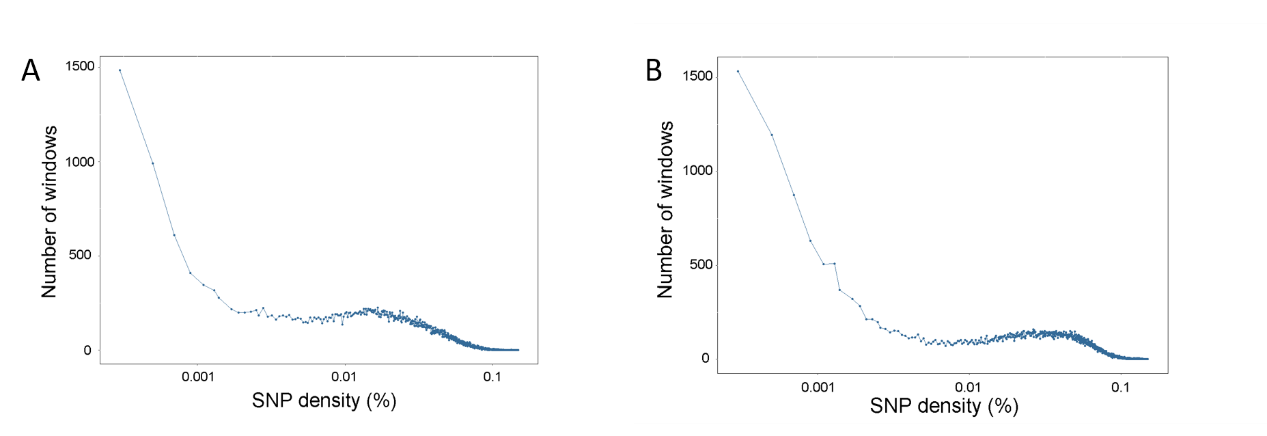


**Supplementary Fig. 10**

Distribution of heterozygosity density. A total of 7.4 / 9.5 M heterozygous SNPs were identified between the two sets of chromosomes of the swamp buffalo (A) / river buffalo (B) diploid genomes. Non-overlapping 50 kb windows were chosen and the heterozygosity density in each window was calculated.


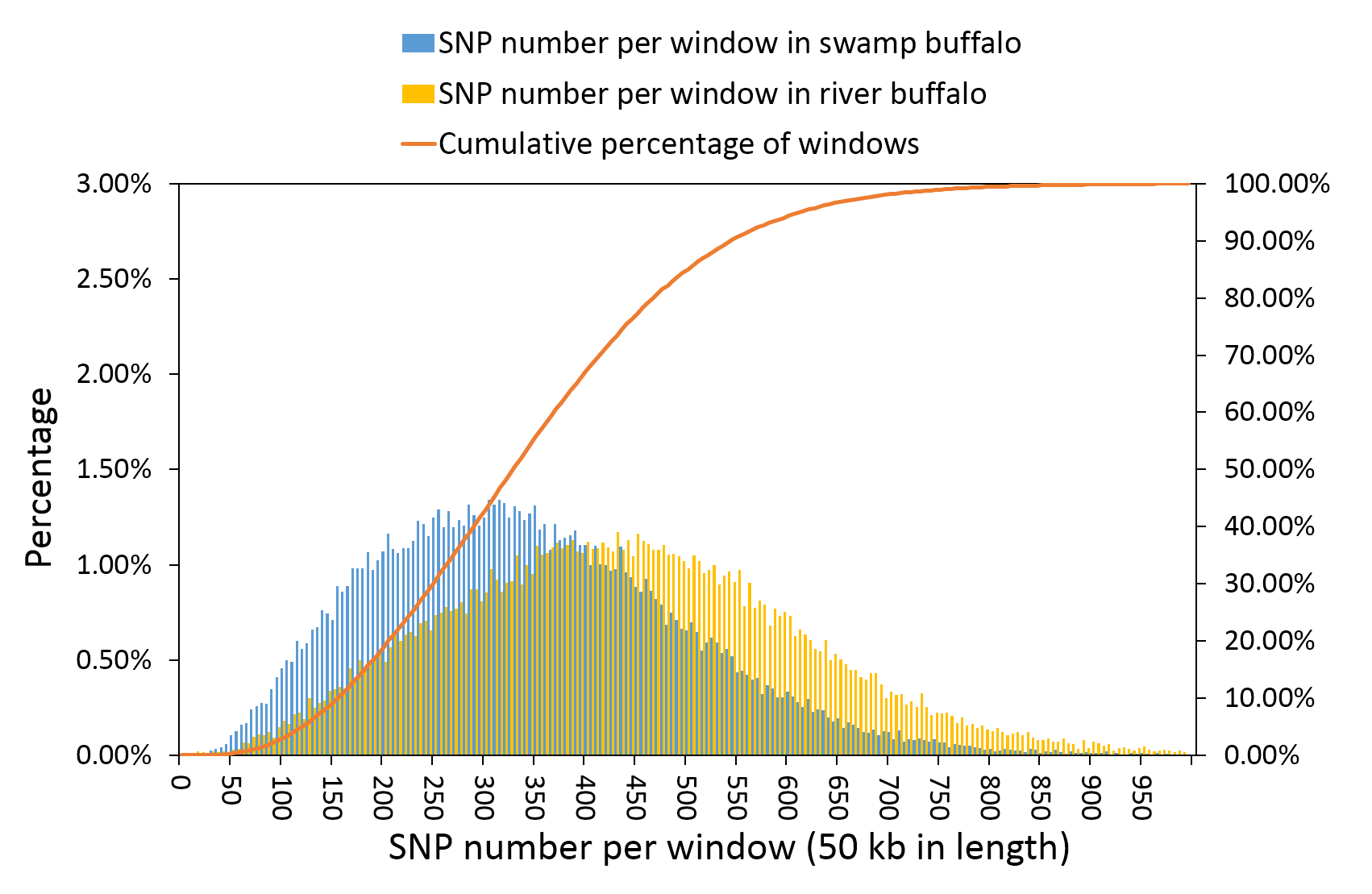


**Supplementary Fig. 11**

Genome-wild distribution of SNPs.


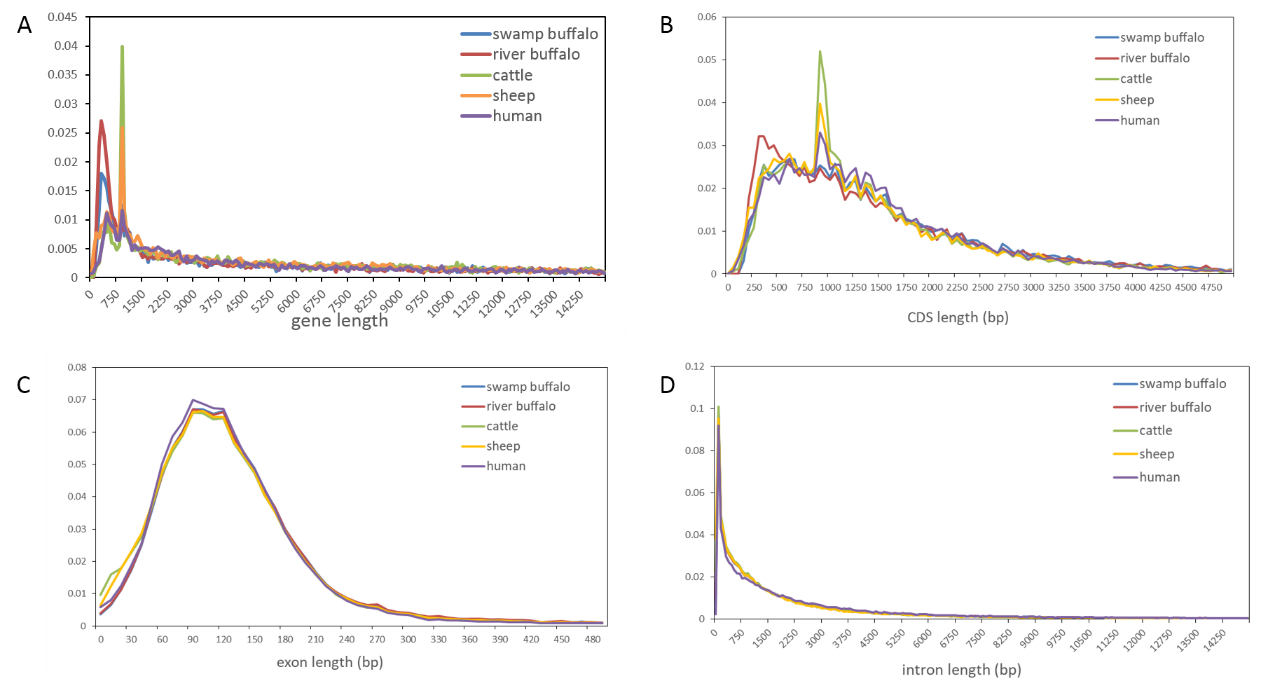


**Supplementary Fig. 12**

Expansion and contraction of gene families among 10 mammal animals. The number of expanded (red) and contracted (blue) gene families are shown on the nodes of tree.


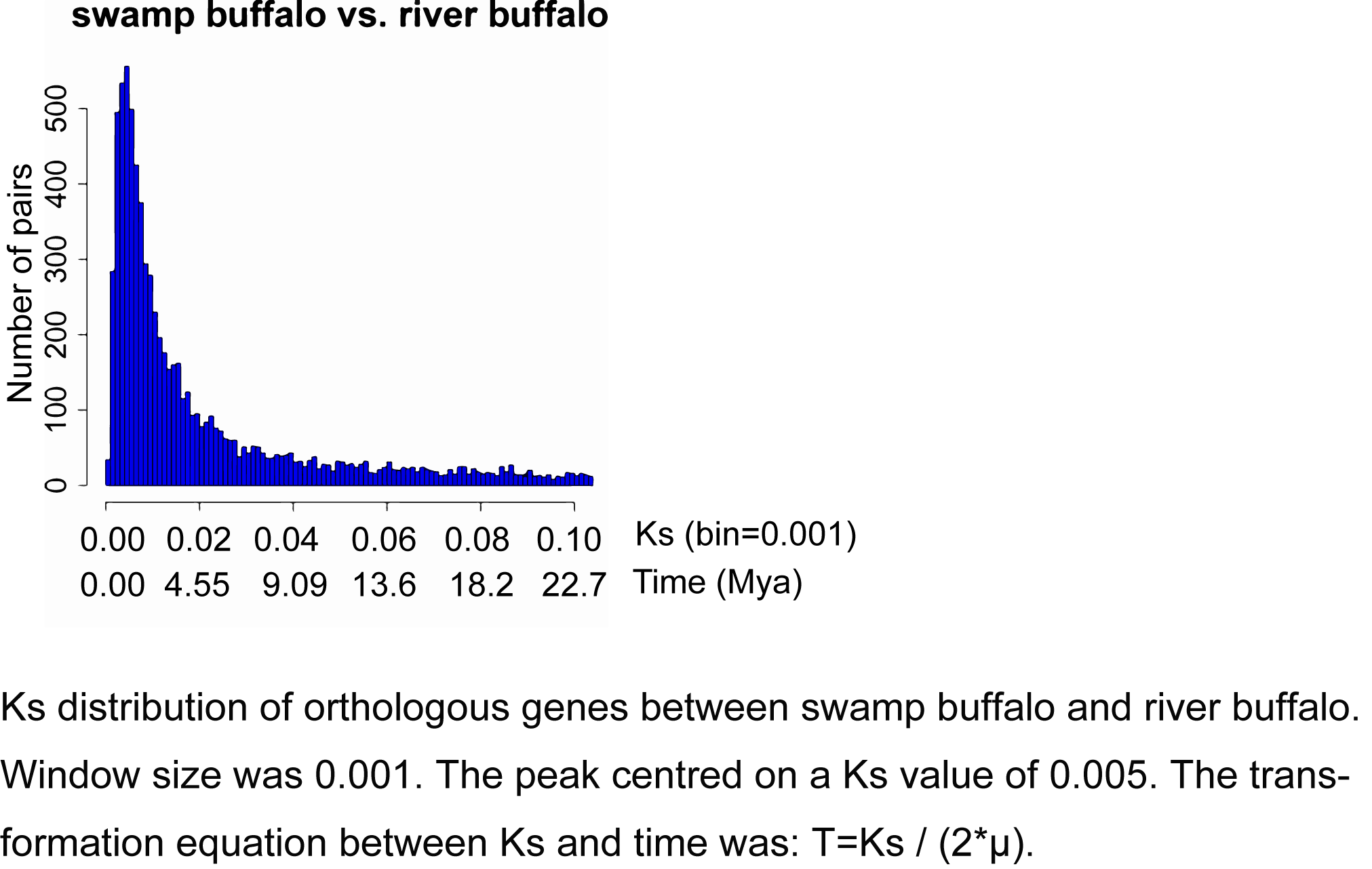


**Supplementary Fig. 13**

Ks distribution of orthologous genes between swamp buffalo and river buffalo. Window size was 0.001. The peak centred on a Ks value of 0.005. The transformation equation between Ks and time was: T=Ks / (2*µ).


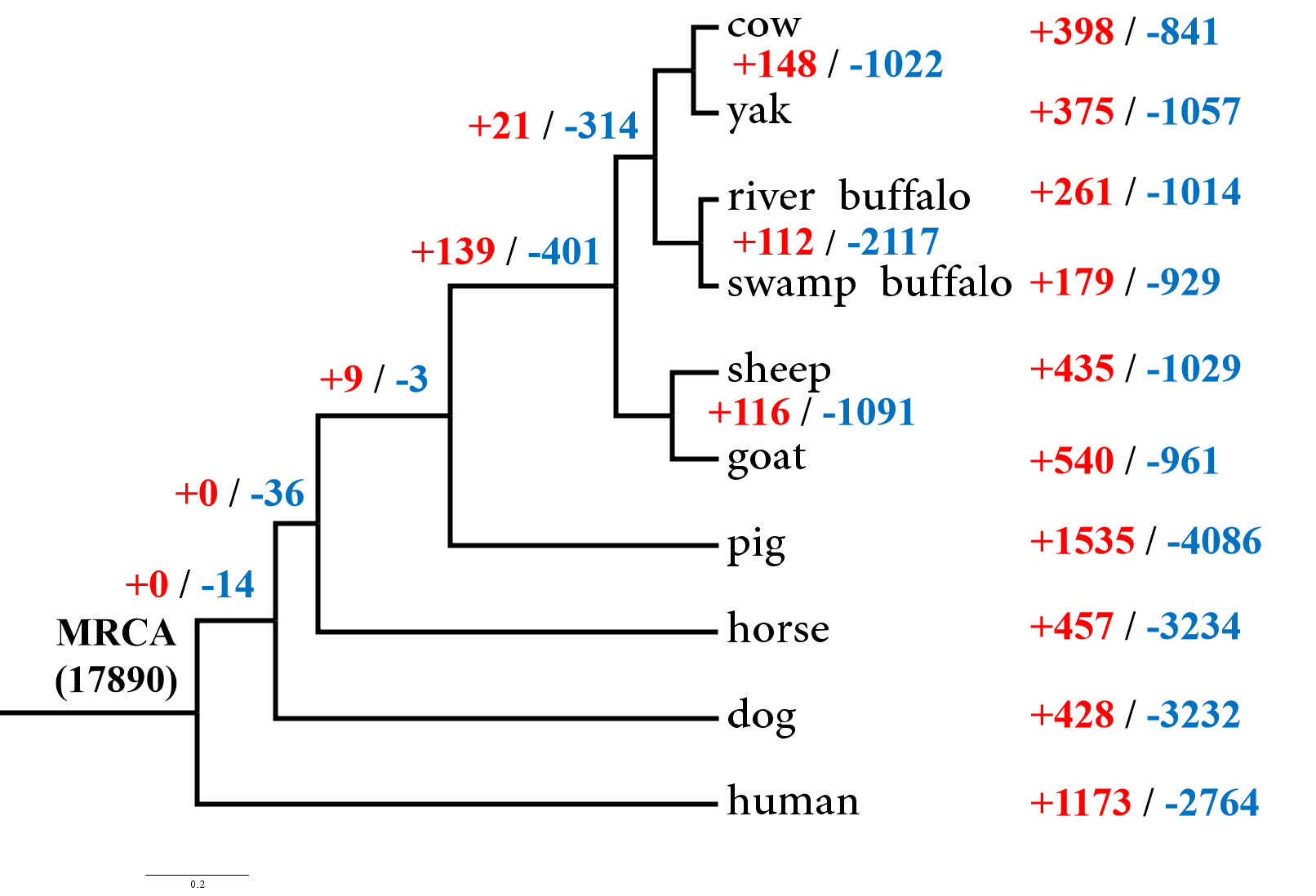


**Supplementary Fig. 14**

Expansion and contraction of gene families among 10 mammals. The number of expanded (red) and contracted (blue) gene families are shown on the nodes of tree.


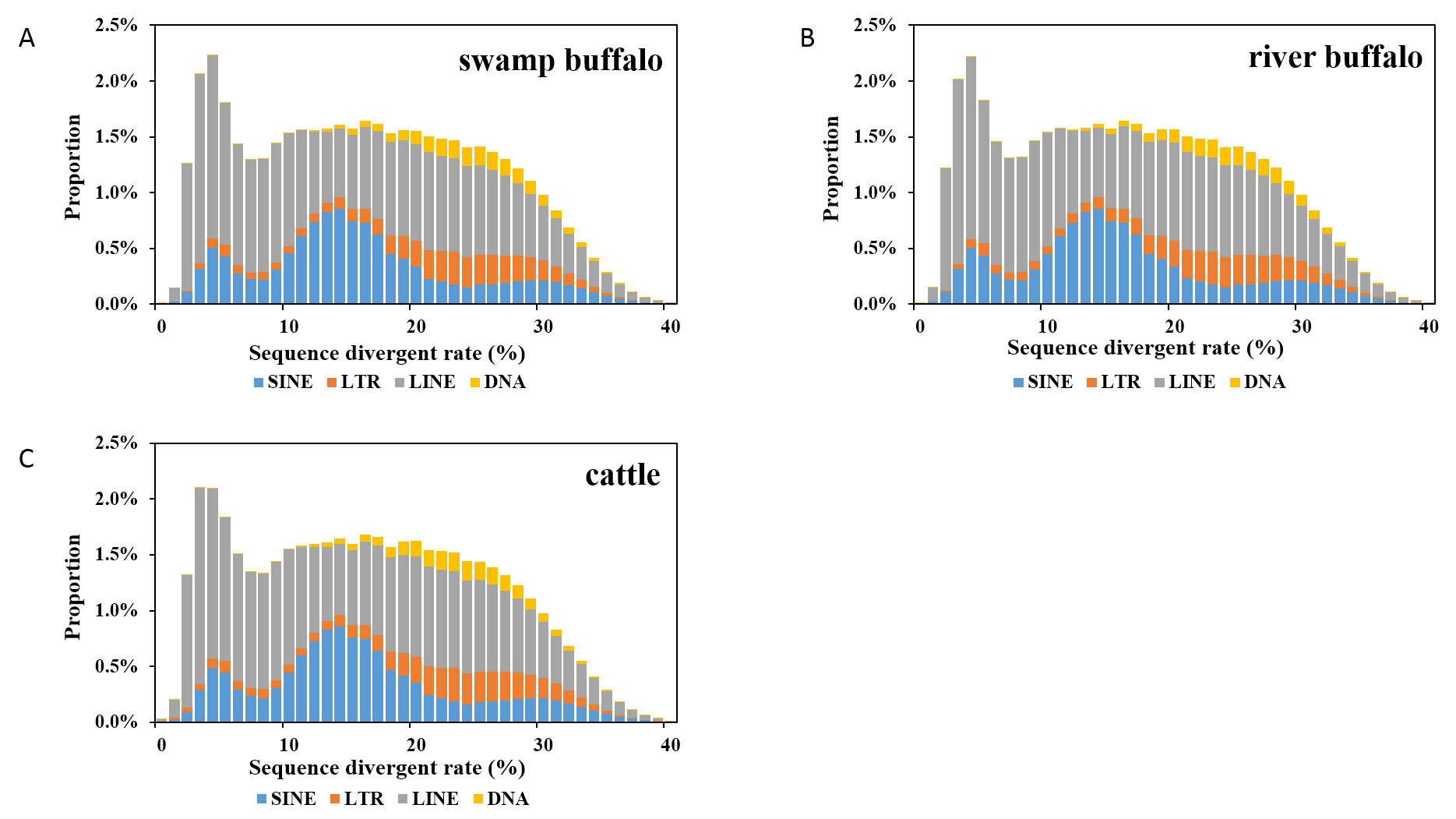


**Supplementary Fig. 15**

Divergence distribution of classified families of transposable elements. The classified transposon families in swamp buffalo (A), river buffalo (B), and cattle (C) genomes were aligned onto the consensus in Repbase. The divergence rate was calculated based on the alignment between the RepeatMasker annotated repeat copies and the consensus sequence in the repeat library.


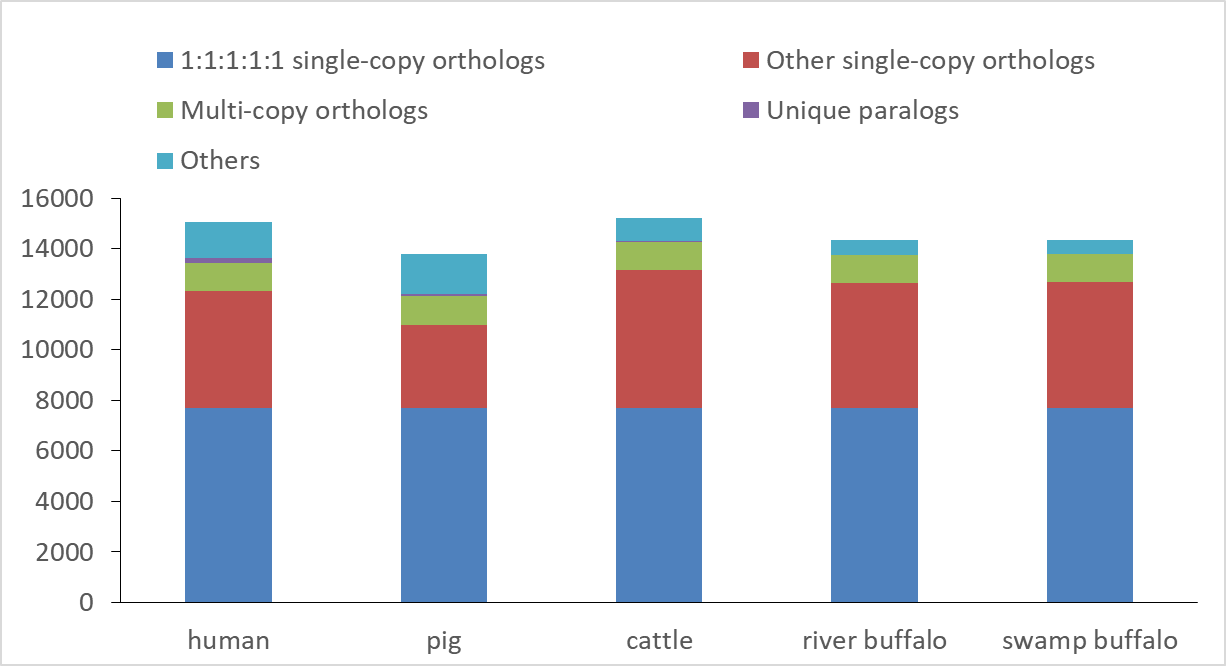


**Supplementary Fig. 16**

Orthology assignment of the swamp buffalo, river buffalo, cattle, pig and human genomes.


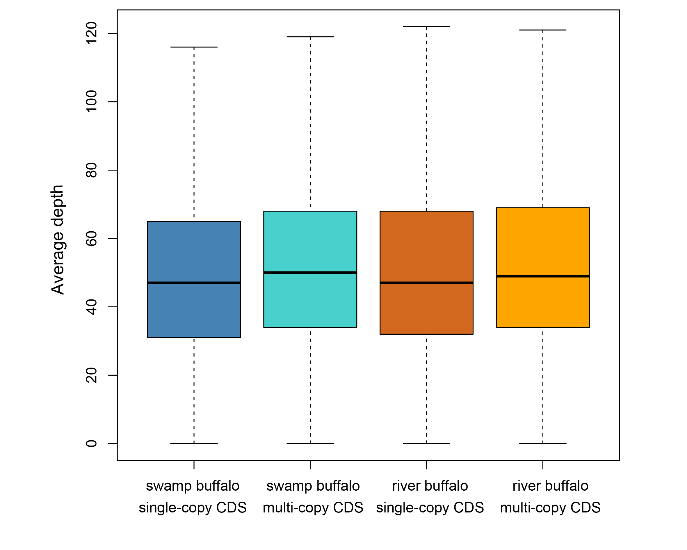


**Supplementary Fig. 17**

Sequence depth distribution between single- and multi-copy genes in the swamp buffalo and river buffalo genome. The whole genome single-base depth was calculated by Samtools and the average depth of each gene only included the CDS region.


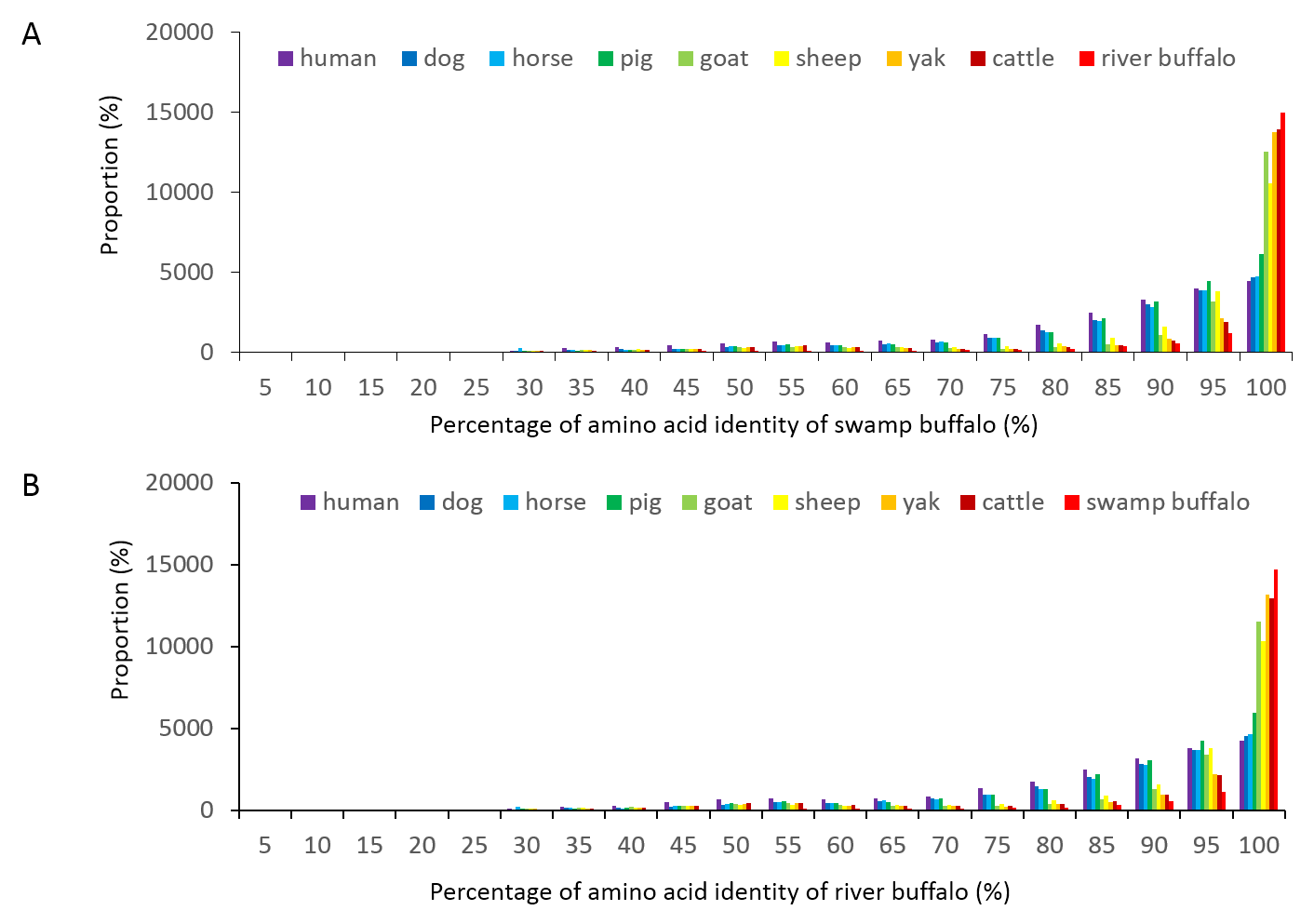


**Supplementary Fig. 18**

Distribution of pairwise amino acid identity of orthologs among ten mammal genomes. A, the identity among swamp buffalo and nine other mammal taxa. B, the identity among river buffalo and nine other mammal taxa.


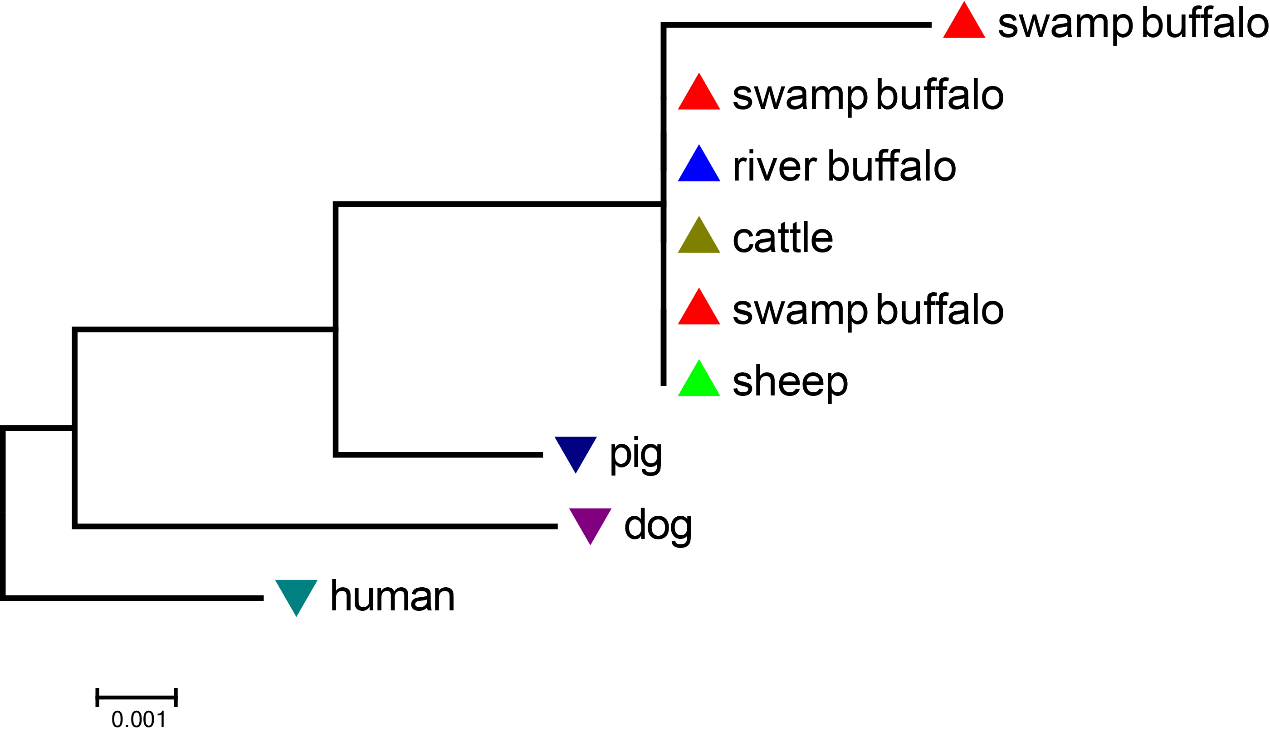


**Supplementary Fig. 19**

Phylogenetic analysis of AMD1 expanded in swamp buffalo. The maximun-likelihood (ML) tree was generated using MEGA 7.0.26.


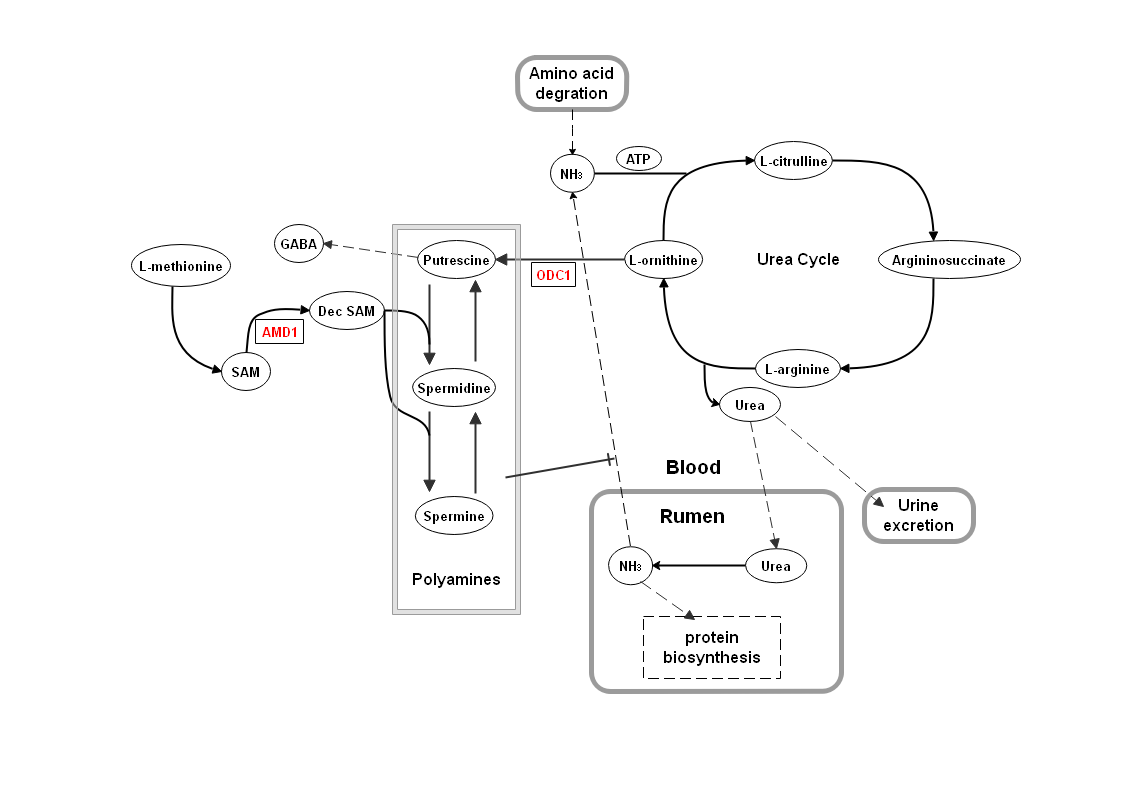


**Supplementary Fig. 20**

The relationship between polyamine metabolism and urea metabolism. SAM, S-adenosylmethionine; Dec SAM, decarboxylated S-adenosylmethionine.


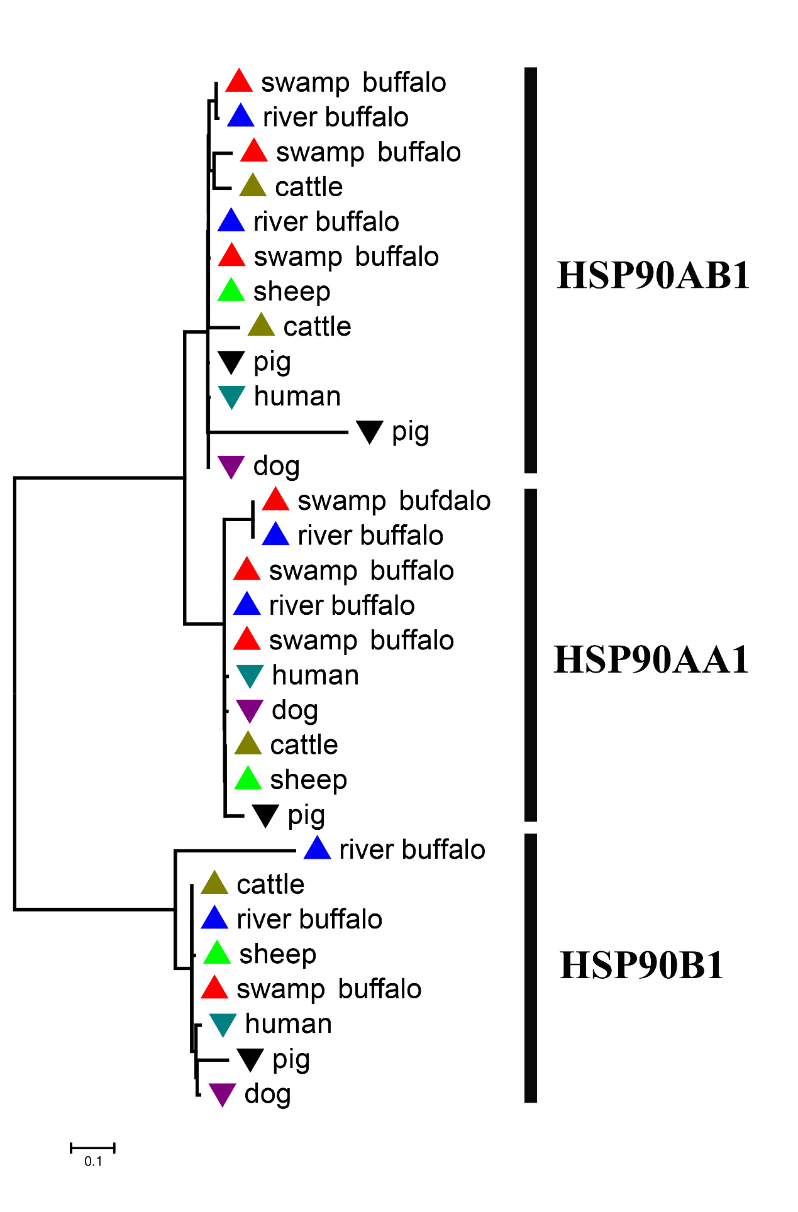
**Supplementary Fig. 21**

Phylogenetic analysis of HSP90 family expanded in both sub-species of buffalo. The maximun-likelihood (ML) tree was generated using MEGA 7.0.26.


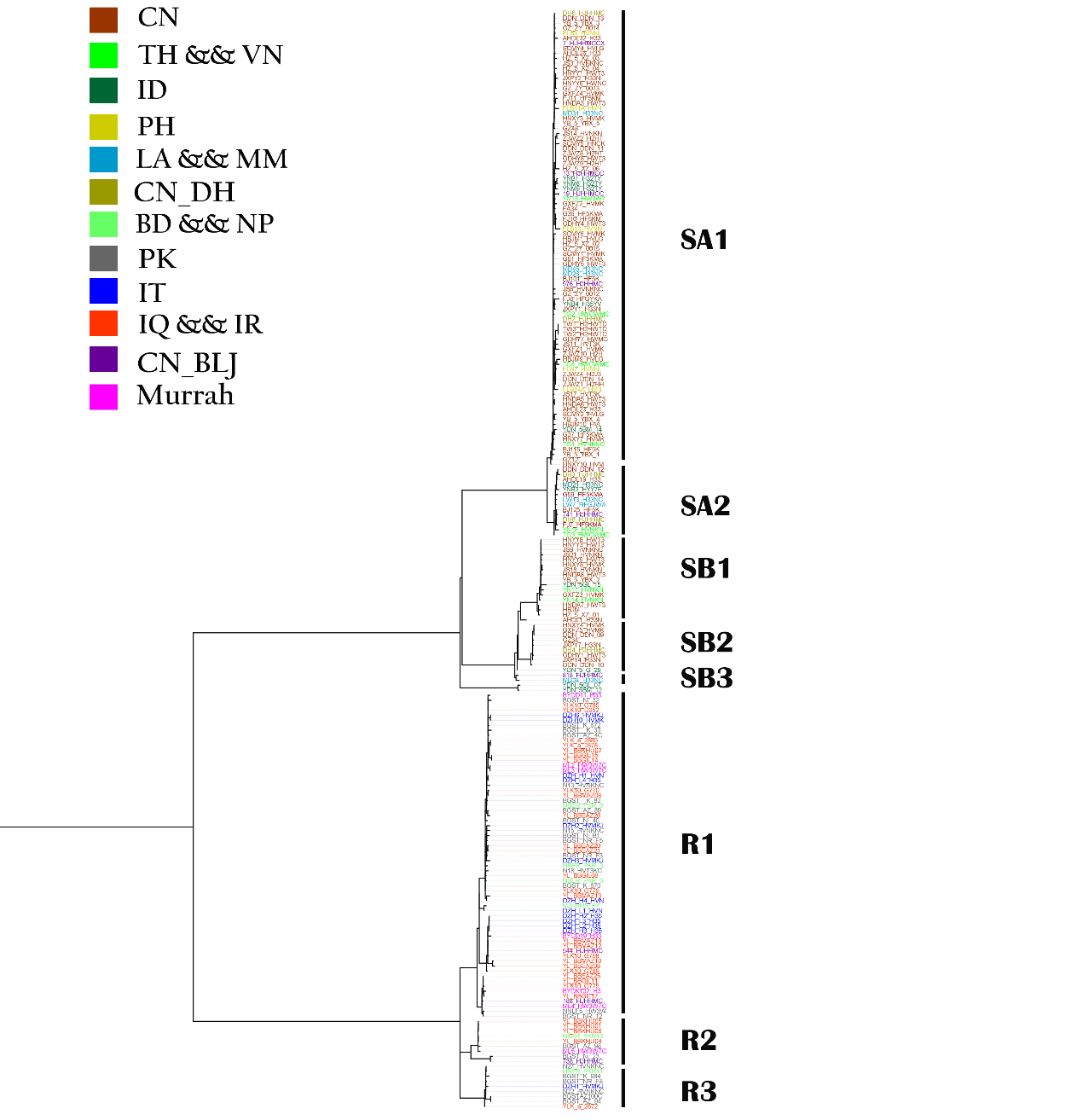


**Supplementary Fig. 22**

Phylogenetic tree inferred from 219 complete mitochondrial genomes. Maximun-likelihood (ML) tree inferred using RAxML 8.2.12. Colour reflected the countries of the sampling. SA and SB represented clades in swamp buffalo. R1, R2 and R3 represented clades in river buffalo.


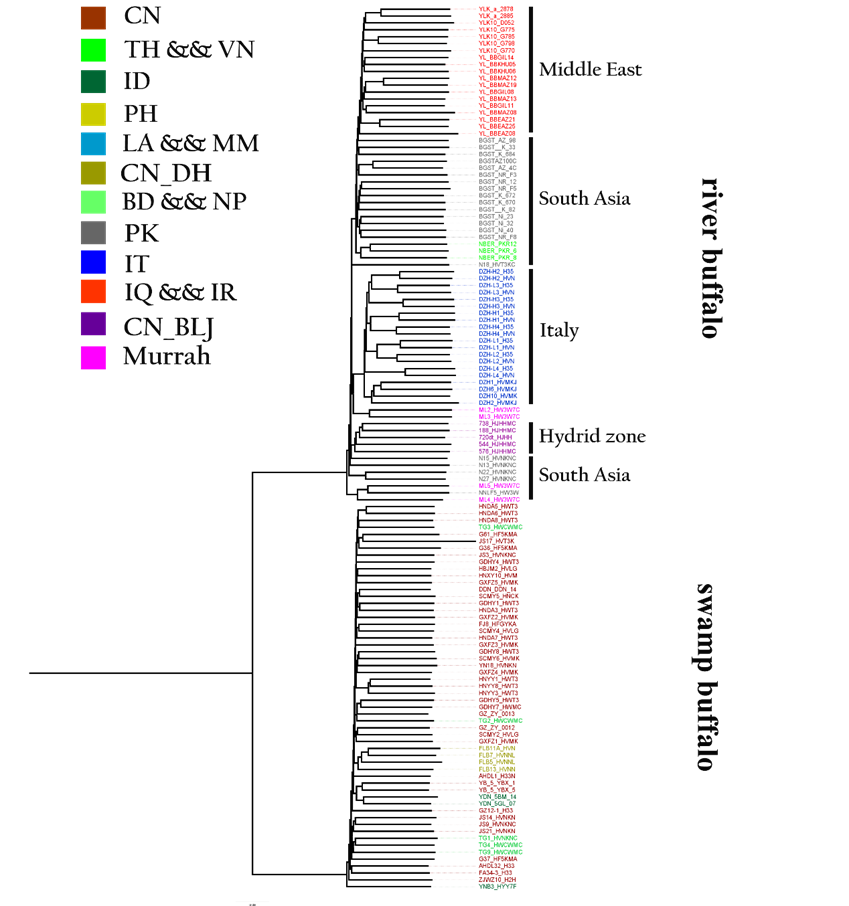


**Supplementary Fig. 23**

Phylogenetic tree inferred from 128 female buffaloes. Neighbour-joining tree (NJ) tree inferred from PHYLIP 3.696. All SNP on the X-chromosome were used. Colour reflected the countries of the sampling. The black lines represented the sampling sites or the original sites. The topological structure among swamp buffalo populations were ambiguous, while the topological structure among river buffalo populations clearly clustered by regions.


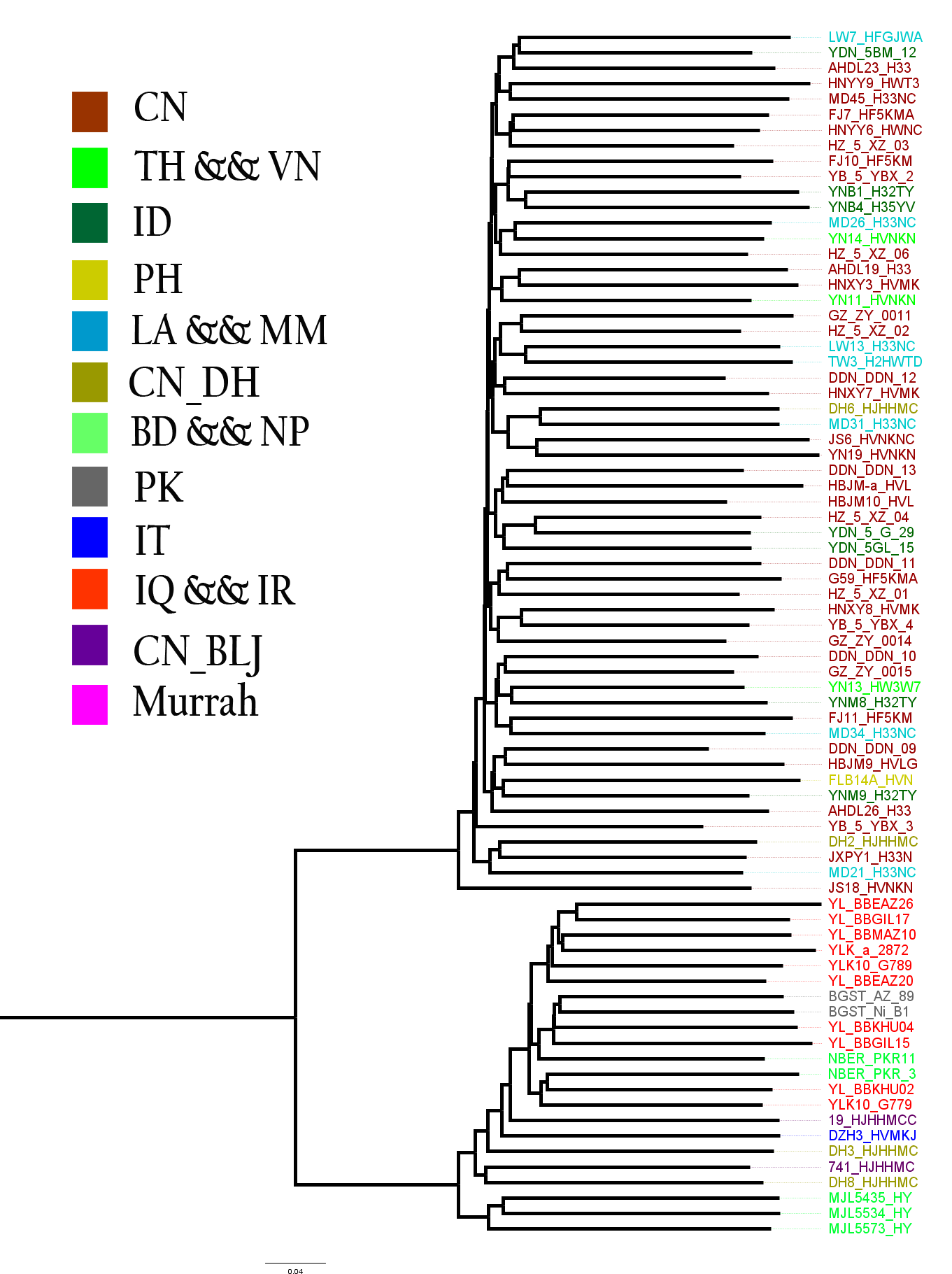


**Supplementary Fig. 24**

Phylogenetic tree inferred from 78 Y-chromosomes in buffalo bulls . The reference used here is the Y chromosome of cattle (Btau 5.0.1). Colour reflected the countries of the sampling.


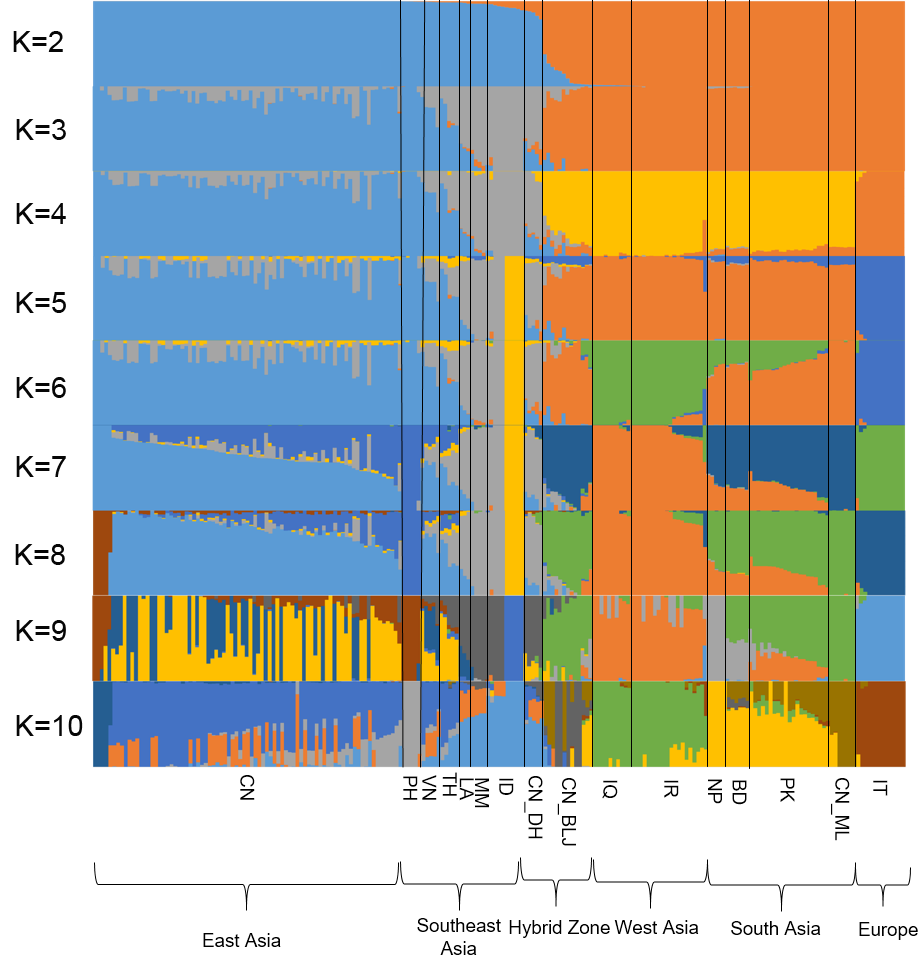


**Supplementary Fig. 25**

Population structure plots with K=2-10.


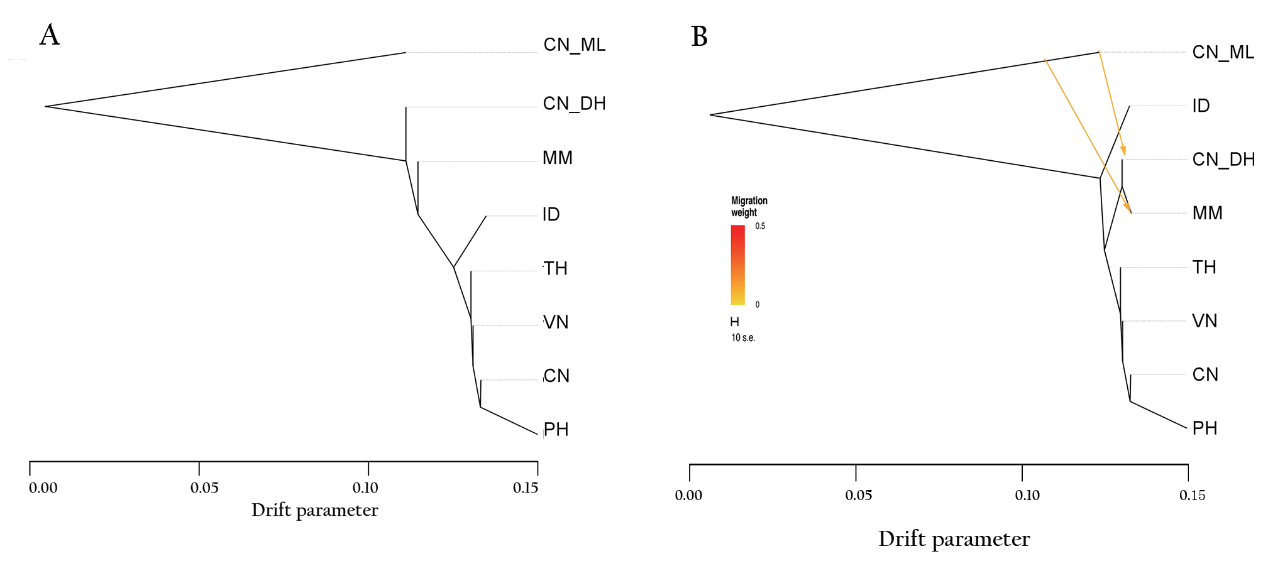


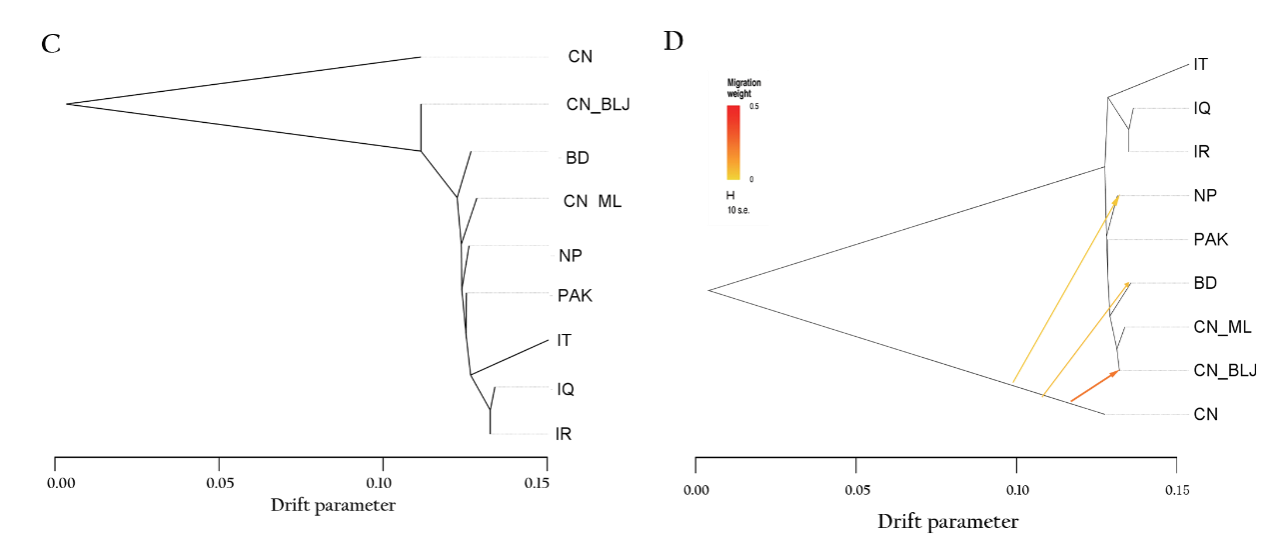


**Supplementary Fig. 26**

Tree topology inferred from TreeMix. (A) Swamp buffalo populations with no migration allowed (B) Swamp buffalo population with 2 migration allowed (C) River buffalo population with no migration allowed (D) River buffalo population with 3 migration allowed.


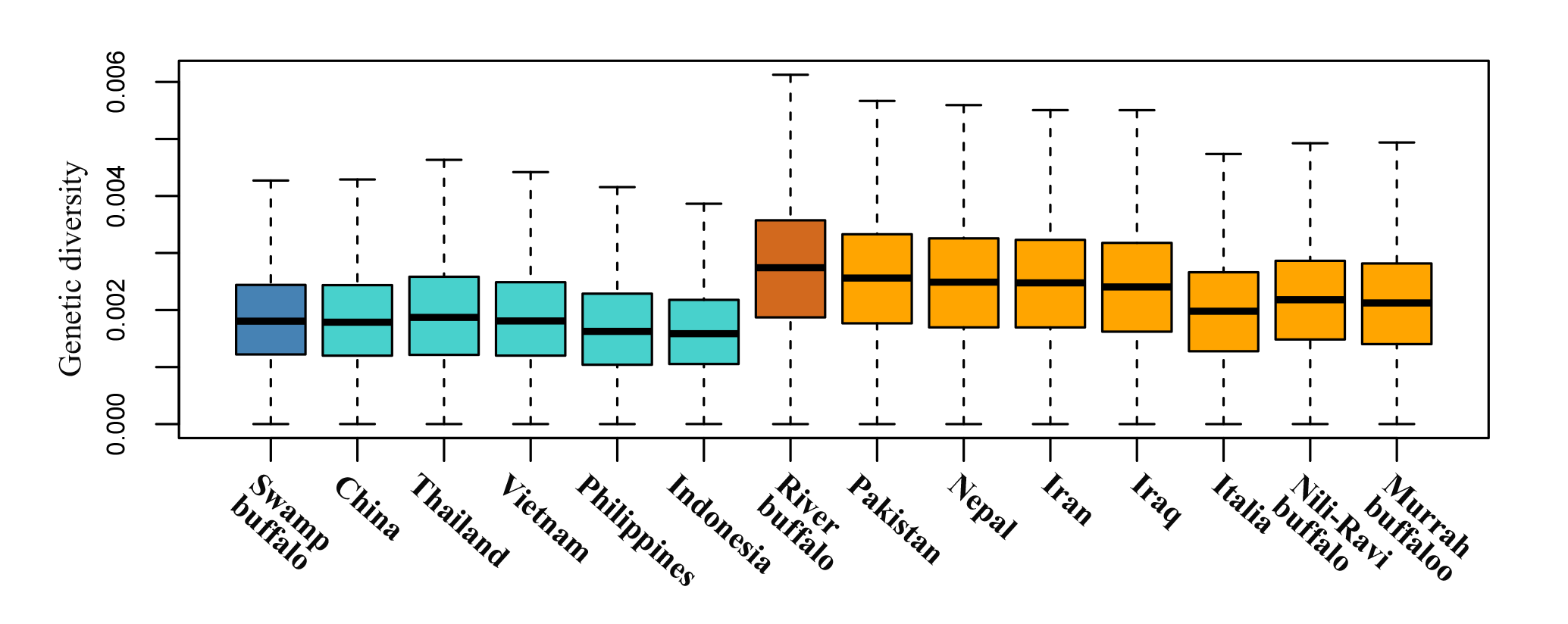


**Supplementary Fig. 27**

Genetic diversity (θ, 4 Nμ) for buffalo populations clustered mainly by region.


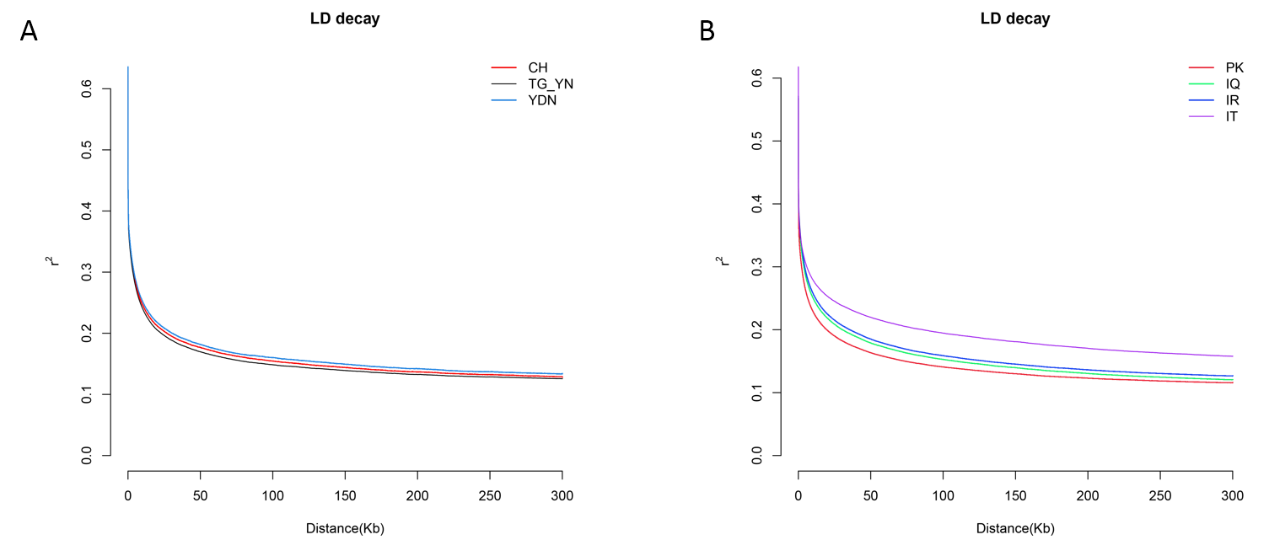


**Supplementary Fig. 28**

Decay of linkage disequilibrium (LD) patterns of the (A) swamp buffalo and (B) the river buffalo.


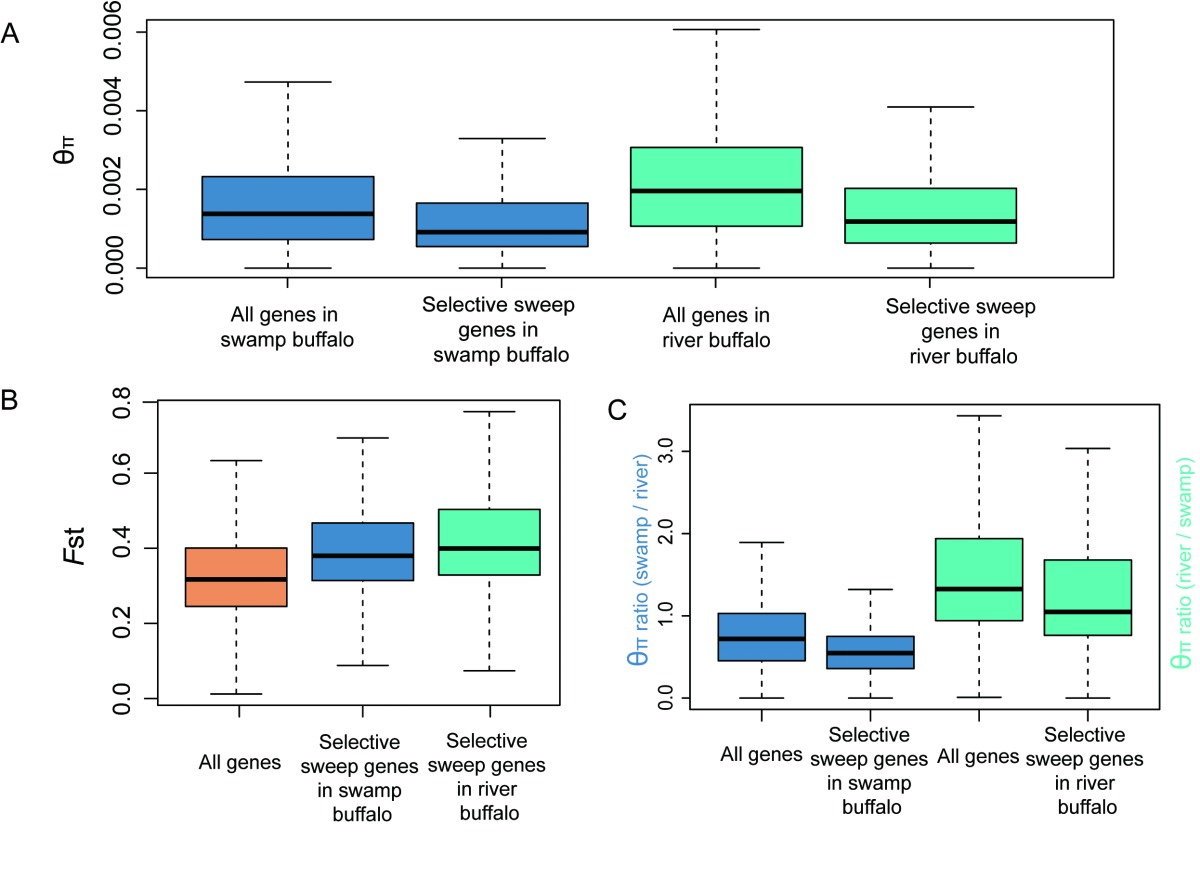


**Supplementary Fig. 29**

Box plot of *θ*π (A), F_ST_ values (B), and *θ*π ratio (*θ*π, swamp/ *θ*π, river; *θ*π, river/ *θ*π, swamp) (C) for selective sweep genes of swamp and river buffaloes versus the whole genome.


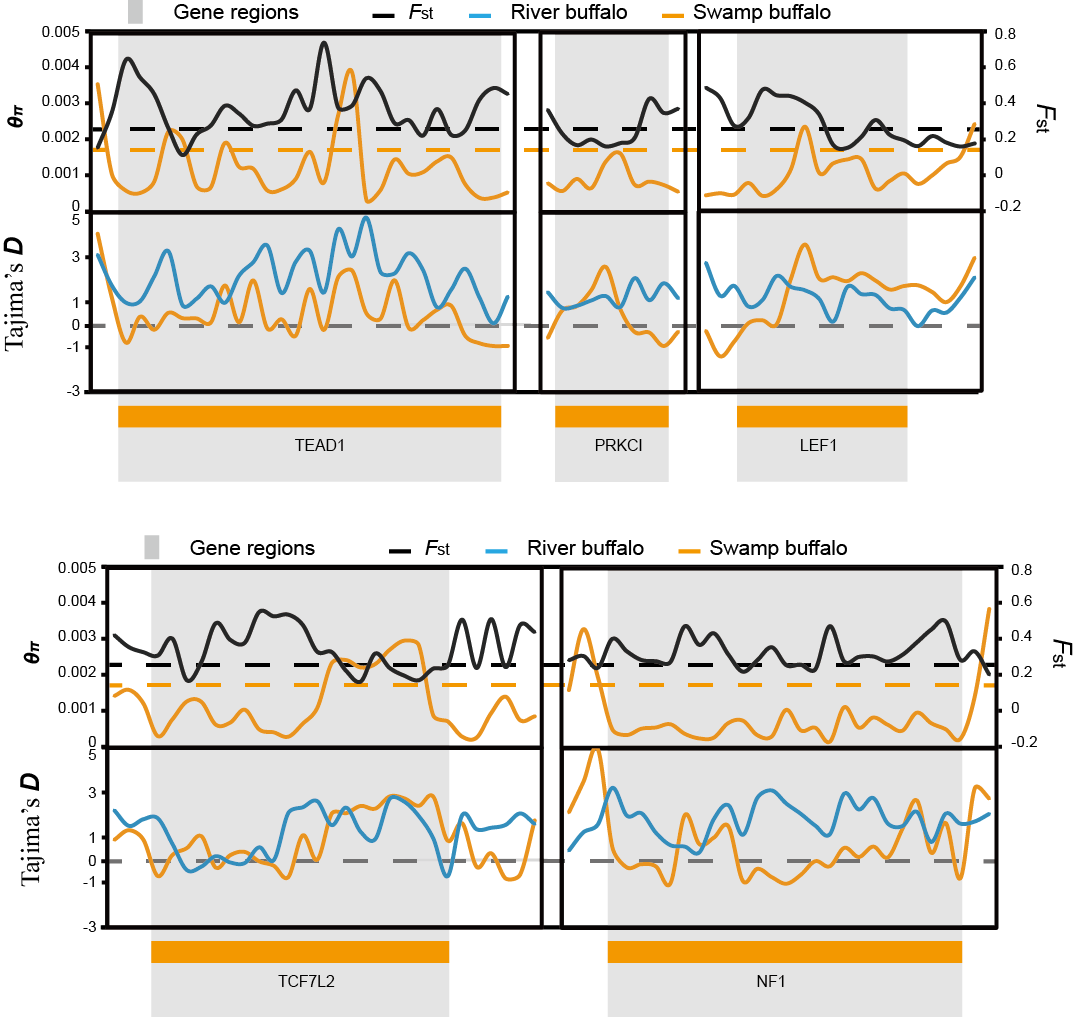


**Supplementary Fig. 30**

Example of genes with strong selective sweep signals in swamp buffalo. The *θ*π, *F*st, and Tajima’s *D* values were plotted with 10kb sliding window. The dark horizontal dotted lines represent the mean value of *F*st; the orange horizontal dotted lines represent the mean value of *θ*π; the grey at the range horizontal dotted lines represent the value of zero.


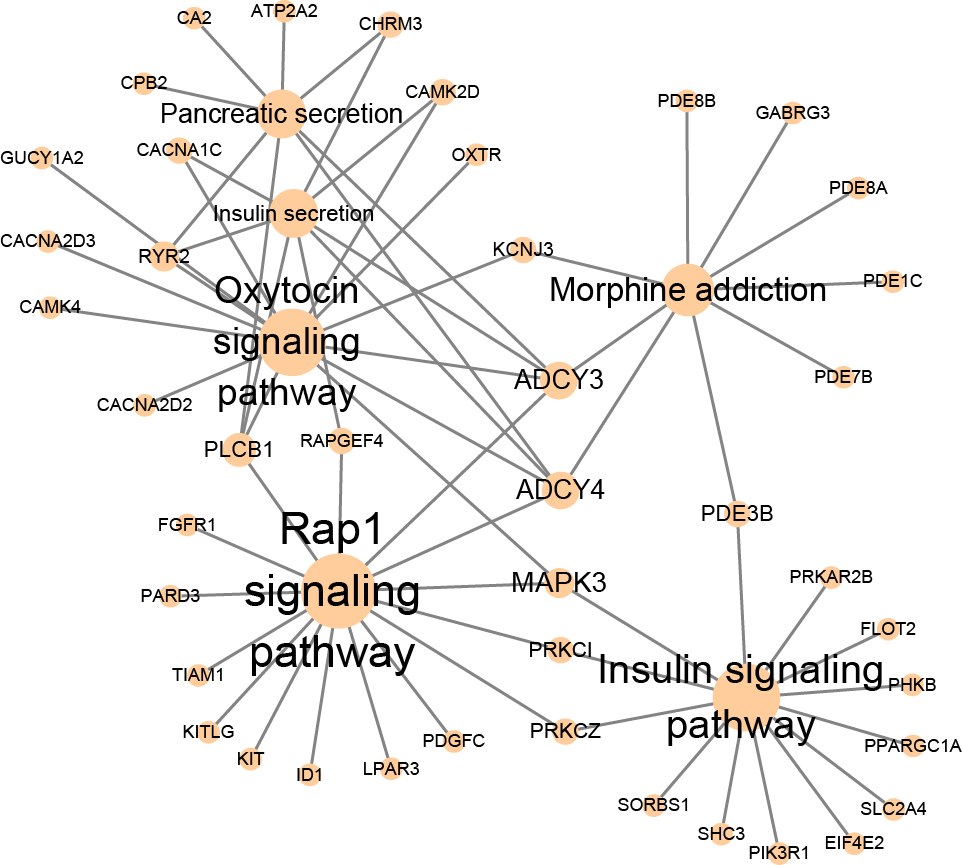


**Supplementary Fig. 31**

KEGG pathways transducted by ADCY3 in the selective sweep genes of swamp buffalo


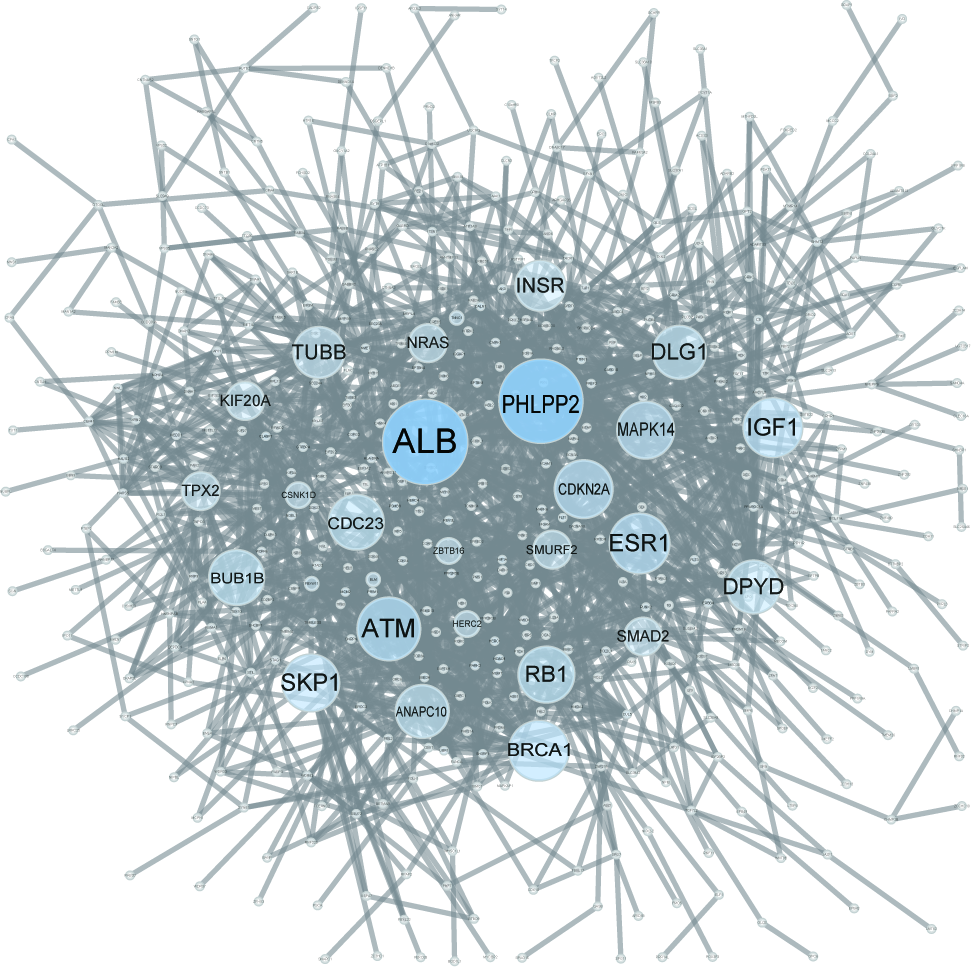


**Supplementary Fig. 32**

Selective sweep genes of river buffalo which display in protein-protein interactive (PPI) network analysed by STRING database. The nodes size and transparency direct correlation to the connection nodes degree.


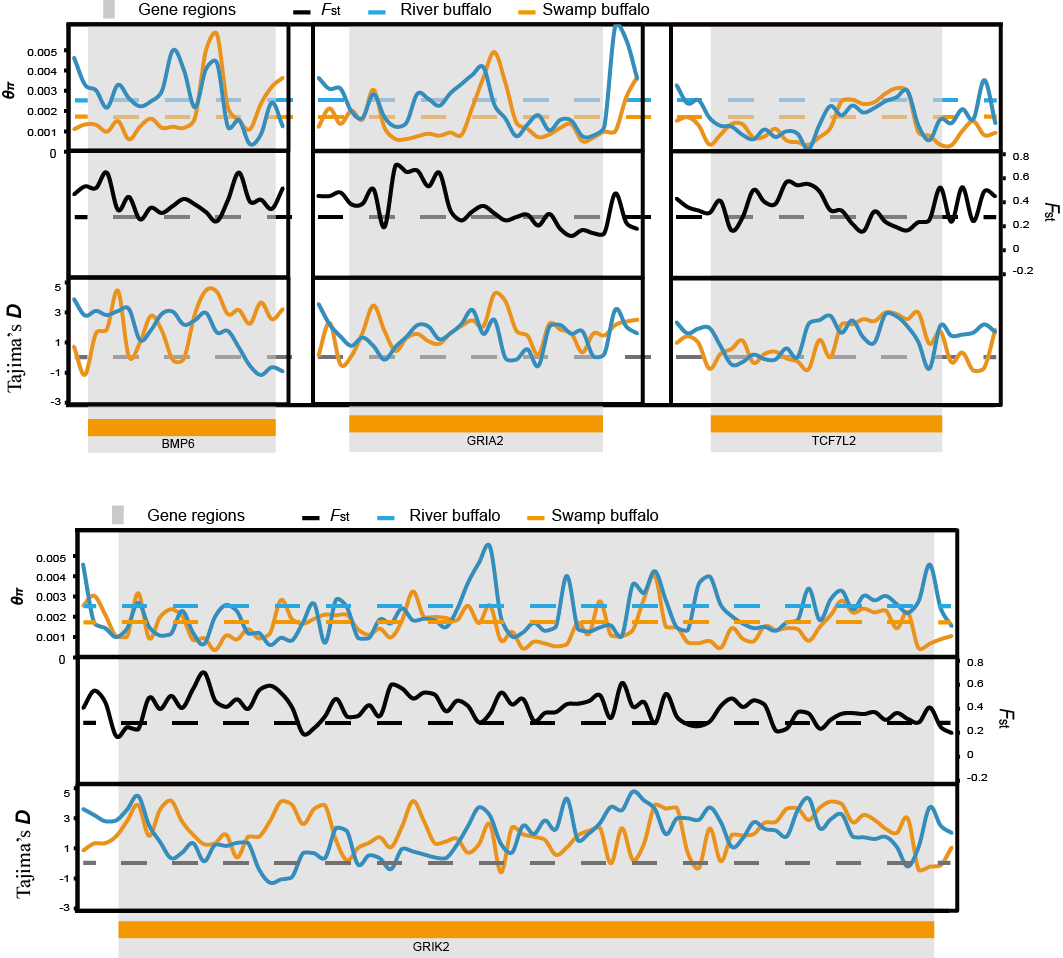


**Supplementary Fig. 33**

Example of genes with strong selective sweep signals of overlapping genes between swamp buffalo and river buffalo. The *θ*π, *F*st, and Tajima’s *D* values were plotted with 10kb sliding window. The dark horizontal dotted lines represent the mean value of *F*st; the orange horizontal dotted lines represent the mean value of *θ*π; the grey at the range horizontal dotted lines represent the value of zero.
