# Supplementary material for: Understanding divergent domestication traits from the whole-genome sequencing of swamp- and river-buffalo populations
Source: Natl Sci Rev. 2020 Feb 17;7(3):686–701. doi: 10.1093/nsr/nwaa024 (PMC8289072; doi:10.1093/nsr/nwaa024)
Supplement: nwaa024_Supplemental_Files [file nwaa024_supplemental_files.zip › Responses-to-the-reviewers'_comments.docx]

**Point-by-point responses to editorial requests**

03-Feb-2020

Dear Prof. Liu,

Manuscript ID NSR_MS-2019-458.R1 entitled "Understanding divergent domestication traits from the whole-genome sequencing of swamp and river buffalo populations" which you submitted to the National Science Review, has been reviewed. The comments of the reviewer(s) are included at the bottom of this letter.

The reviewers have recommended publication, but also suggested some minor revisions to your manuscript. Therefore, I invite you to respond to the reviewer(s)' comments and revise your manuscript.

Please make sure to polish the language but we will accept it.

******

https://mc.manuscriptcentral.com/nsr_ms?URL_MASK=52fcca44663f488c8398dc1f3ed046a4

******

Once again, thank you for submitting your manuscript to the National Science Review and I look forward to receiving your revision.

Sincerely,

Dr. Xiuling Xu

Managing Editor, National Science Review

xuxiuling@scichina.org

Reviewer(s)' Comments to Author:

**Reviewer: 1**

Comments to the Author

The authors revised the manuscript accordingly. I have no more comments in science, and recommend to be accepted by several minor revisions as follows:

Q1. Figure 3 and Supplementary Fig. 33, draw the significant/cutoff line.

A1: Thanks for the reviewer’s suggestion. It was different to the method of identifying selective sweep genes (*θ*π, sweepfinder2, iHS and recombination rate) and validating genes in figure3 (*θ*π, Fst, and Tajima’s D). It was hard to represent the 6 parameters in the figures. We added the cutoff values of *θ*π and iHS in the method at line 598-605.

Q2. Use the Scientific notation on numbers, for instance line 164-165, 2117, 1022 to 2,117, 1,022; and line 534, P = 8.968E-13 to P = 8.97E-13.

A2: Thanks for the reviewer’s suggestion. We followed the reviewer’s suggestion and edited them.

**Reviewer: 2**

Comments to the Author

Q3. The authors have addressed my questions. I highly recommend the authors to find a native English speaker to correct the English presentation.

A3: Thanks for the reviewer’s suggestion. We invited two English speakers to polish the presentation and combined their suggestion. The modification was highlighted in the manuscript.

**Reviewer: 3**

Comments to the Author

The authors answered all my concerns and I have no further comments on it.
